# The Effectiveness of Co-Inoculation by Consortia of Microorganisms Depends on the Type of Plant and the Soil Microbiome

**DOI:** 10.3390/plants13010116

**Published:** 2023-12-31

**Authors:** Ekaterina Alexeevna Sokolova, Olga Viktorovna Mishukova, Inna Viktorovna Hlistun, Irina Nikolaevna Tromenschleger, Artem Yurievich Tikunov, Andrey Dmitrievich Manakhov, Evgeny Ivanovich Rogaev, Oleg Alexandrovich Savenkov, Maria Dmitrievna Buyanova, Ilya Vladimirovich Ivanov, Natalya Valentinovna Smirnova, Elena Nikolaevna Voronina

**Affiliations:** 1 Institute of Chemical Biology and Fundamental Medicine, Siberian Branch of the Russian Academy of Sciences, 630090 Novosibirsk, Russia; mishukova_olga@inbox.ru (O.V.M.); inna.kotliarova@yandex.ru (I.V.H.); irina510@ngs.ru (I.N.T.); arttik1986@gmail.com (A.Y.T.); 2 Department of Natural Sciences, Novosibirsk State University, 630090 Novosibirsk, Russia; 3 Department of Genetics, Centre for Genetics and Life Science, Sirius University of Science and Technology, 354340 Sirius, Russia; manakhov@rogaevlab.ru (A.D.M.); evivrecc@gmail.com (E.I.R.); 4 Institute of Soil Science and Agrochemistry, Siberian Branch of Russian Academy of Sciences, 630090 Novosibirsk, Russia; savenkov@issa-siberia.ru (O.A.S.); sssadm00n@yandex.ru (M.D.B.);

**Keywords:** consortia of microorganisms, co-inoculation, soil microbiom

## Abstract

The amalgamation of mineral and targeted bacterial preparations represents a new generation of agricultural technology. Inoculation with combined preparations of microorganisms is more effective than inoculation with a single microorganism in stimulating plant growth by providing a more balanced diet for various crops. In this work, the effect of inoculation of 20 consortium variants on the yield indicators of three crops (wheat, buckwheat, corn) and the soil microbiome in the open field was investigated. The soil microbiome was defined by 16S rRNA sequences through NGS. The species richness of the soil microbial community (alpha diversity) was similar for all studied samples. A beta-diversity analysis revealed that the microbial diversity of three soil samples (C.bw, F.bw and Soil.bw) differed significantly from all others. At the phylum level, the number of *Acidobacteriota* and *Firmicutes* in these samples was increased. For the combination “Consortium C (*Rothia endophytic GMG9* and *Azotobacter chroococcum GMG39*)—buckwheat”, a systemic positive improvement in all growth and yield indicators was observed. The soil of the site where buckwheat grew, inoculated by Consortium C, contained significantly more available phosphorus than all other soil samples. Such results can be explained both by the direct action of a consortium of phosphate-immobilizing and nitrogen-fixing bacteria and acidification of the medium due to an increase in phylum *Acidobacteriota* bacteria in the soil.

## 1. Introduction

The yield of plants is directly dependent on the productivity of the interaction between soil, plants, and microorganisms. Through microorganisms, plants not only fulfill their nutrient requirements (nitrogen, phosphorus, potassium, etc.) but also gain protection from phytopathogens. This effect is achieved through various mechanisms, including: (a) the increased mobilization of insoluble nutrients, subsequently enhancing assimilation by plants [1], (b) the production of plant growth hormones like auxins [2], cytokinins [3], gibberellins [4], and (c) antagonism against phytopathogenic microorganisms by producing siderophores [5]. Consequently, microbial preparations can significantly reduce the need for mineral fertilizers, thereby enhancing their efficiency of use. The amalgamation of mineral and targeted bacterial preparations represents a new generation of agricultural technology.

It has been reported that inoculation with combined preparations of microorganisms is more effective than inoculation with a single microorganism in stimulating plant growth by providing a more balanced diet for various crops [6,7]. Zhang Yi et al. demonstrated that the co-inoculation of phosphate-solubilizing bacteria (PSB) and phosphate-accumulating bacteria led to higher levels of microbial biomass phosphorus and polyphosphate [8]. The synergistic effect of *Bradyrhizobium japonicum USDA110* and *Pseudomonas putida NUU8* for soybeans in arid field conditions exhibited a significant increase in root length by 56%, shoot length by 33%, dry root mass by 47%, dry shoot mass by 48%, and the number of nodules by 17% compared to the control [9]. The synergistic effect of microorganisms is observed even if each of them individually exhibits different properties. For example, Wang showed the effect of combined treatment of PSB (*Bacillus megaterium* and *Pseudomonas fluorescens*) and N2-fixing bacteria (*Azotobacter chroococcum* and *Azospirillum brasilence*) on the availability of nitrogen and phosphorus within the first 60 days after the addition of bacteria [10]. In addition, the beneficial effect of PSB on the survival of *Azotobacter* in the rhizosphere was observed [11]. Belimov et al., using the 15N isotope dilution method, showed that combined inoculation significantly increased the accumulation of nitrogen fertilizers in plants. Consequently, N2 fixation is not the main mechanism affecting plant growth reactions, and the effect of joint inoculation on their nitrogen nutrition can be explained by an increase in the extraction of nitrogen fertilizers. It is possible that the effect of bacterial mixtures on the mineral nutrition of plants is due to growth-stimulating substances secreted by bacteria [12]. Thus, microorganisms play an important role in agriculture, promoting the circulation of nutrients in plants and reducing the need for chemical fertilizers, although many questions remain unanswered.

Do the same bacteria have the same effect on different plants? Do microorganisms exhibit properties predicted by laboratory tests in an open field? To address these questions, an experiment was conducted with 20 consortia composed of phosphate-immobilizing and nitrogen-fixing bacteria. The experiment involved three plants belonging to the main agricultural areas of plant cultivation in Western Siberia: cereals—wheat, cereals—buckwheat, and green biomass—corn.

## 2. Results

### 2.1. Yield of Buckwheat, Wheat, and Corn

**Figure 1:**
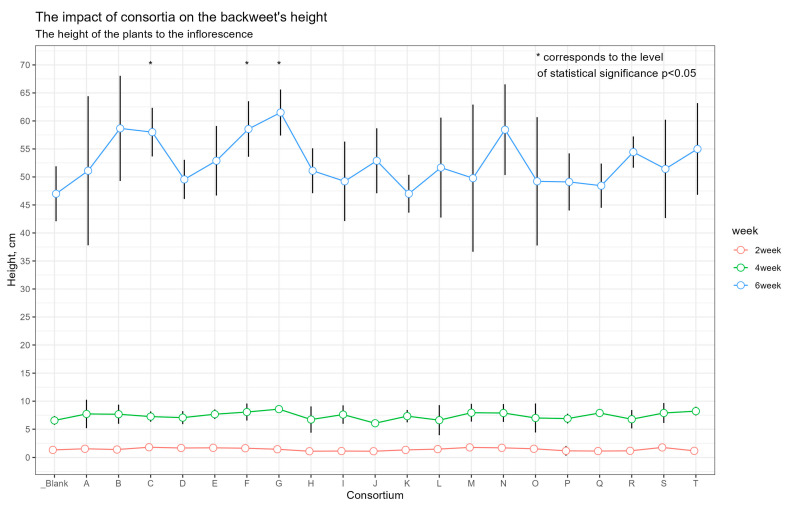
Buckwheat’s height at 2, 4 and 6 weeks. The red line corresponds to 2 weeks of growing, the green one to 4 weeks, and the blue one to 6 weeks of growing. Dots are the mean; whiskers are the SD. Orange line corresponds the mean height of control plants after 6 weeks; the blue ones correspond to SD.

In the initial weeks of growth, buckwheat plants were approximately the same height in all plots, but after 6 weeks, plants from plots C (23.4%), F (24.6%) and G (30.9%) showed a noticeable increase in height versus the control, with a statistical significance level of *p* < 0.05 (Figure 1, Supplementary Table S1).

**Figure 2:**
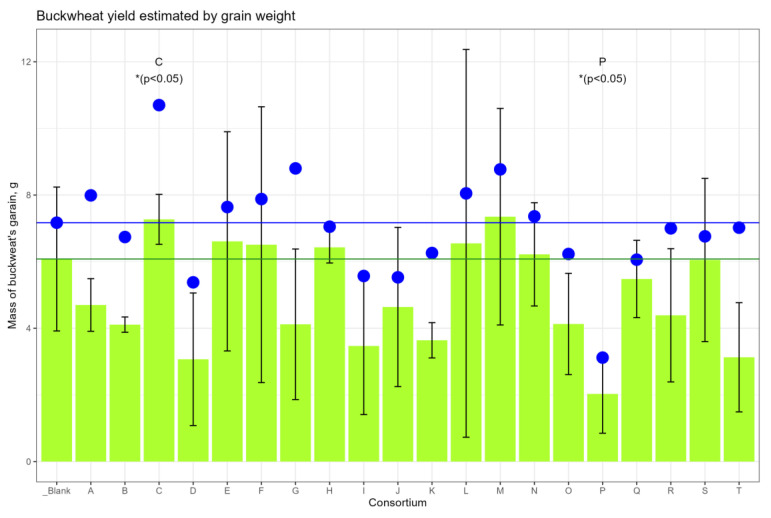
Buckwheat’s grain yield. The height of the green columns corresponds to the mean weight of grain from three plots; the whiskers are SD. The green horizontal line shows the mean weight from the control plot. The blue dots represent the average weight of the grain from the plot, taking into account the grain fallen to the ground. The blue horizontal line corresponds to the dot of the control plot.

The average amount of ripe grain harvested from the ears, compared with the control site, was higher in the sites corresponding to consortia C, E, F, H, L, M and N, and less in the site with consortium P (Figure 2); however, the differences did not reach the level of statistical significance. Taking into account the fallen grain, the yield change reached the level of statistical significance (*p* < 0.05) for consortia C (+49.2%) and for consortium P (−56.5%) (Supplementary Table S2).

**Figure 3:**
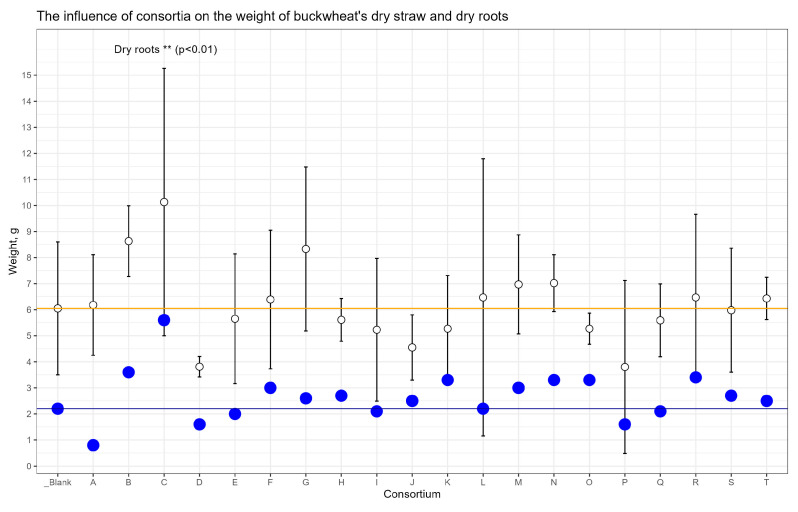
The influence of consortia on weight of buckwheat dry straw and dry roots. The white dots are the mean weight of dry straw, whiskers are the SD. The blue dots are the mean weight of dry roots. The blue line corresponds to the mean weight of dry roots from the Blank plot. The orange line corresponds to the mean weight of dry straw from Blank plot.

The consortium’s influence on the weight of dry straw and dry roots was also revealed (Figure 3). An increase in the mass of dry straw for Consortia B, C, and G was observed, but the threshold of statistical significance was not reached. The weight of dry roots was dramatically strong for consortium C (+152%, *p* < 0.01) versus control plants.

Thus, based on agronomic measurements, it was found that buckwheat plants grown from seeds inoculated with Consortium C consistently outperformed control plants in terms of their indicators: the growth rate, the mass of the vegetative part, and the mass of the grain.

There was no difference between the height of wheat plants in different consortia compared to the control after either 4 weeks or 6 weeks (Supplementary Table S3 and Figure S1). Despite the fact that the stems of plants from the consortium at the site were statistically significantly longer than those of the control, after 3 months (Supplementary Figure S2), the influence of consortia on the length of the wheat ear, as well as on the ratio of the length of the stem to the length of the ear, was not evident (Supplementary Table S4). Also, no effect was found on such yield characteristics as the weight of the ear and the weight of grain without a floor (Supplementary Figure S3).

To assess the effect of the inoculation of corn seeds in bacterial consortia, plant height was measured after 4 and 6 weeks, and the weight of wet and dry ears and the weight of dry roots were measured after 3 months. However, none of these indicators were statistically significantly different compared to the control plot (Supplementary Table S4, Supplementary Figures S4 and S5).

### 2.2. Soil Nutrient Status

**Figure 4:**
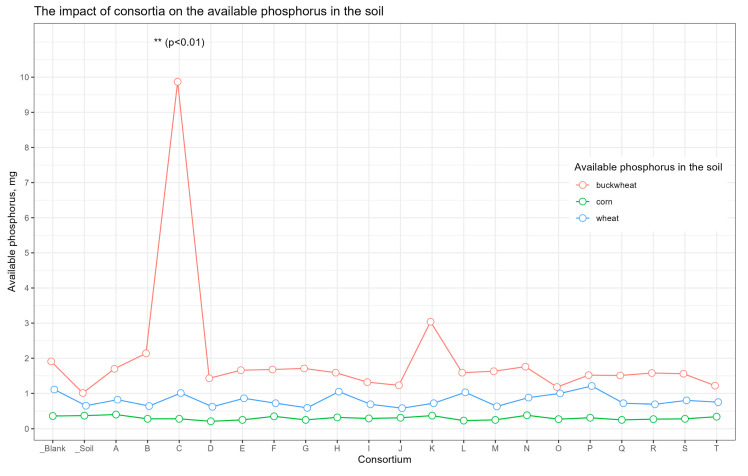
The amount of soluble phosphorus in soils. The red line corresponds to soil samples under buckwheat, the green one to samples under corn, and the blue one to samples under wheat. Dots are the mean equal. **—two asteriks marks the consortia plot, which shows statistical significance differense at level *p* < 0.01.

The analysis of soil nutrients indicated that the content of the available forms of nitrogen, potassium, and carbon (organic, inorganic, and total) was approximately consistent across all samples (Supplementary Table S5). For the soil sample from the plot with buckwheat inoculated in Consortium C, the amount of available phosphorus was significant compared to the other samples (*p* < 0.01) (Figure 4).

### 2.3. Effect of Different Consortium on Soil Microbial Community and Diversity

The dependence of the number of identified taxa on the number of sequences was estimated by constructing rarefaction curves (Supplementary Figure S6). The analysis showed a complete determination of the taxonomic composition, even with 700 sequences in all soil samples, as the curves reached a plateau. The sequencing depth proved adequate to assess alpha diversity.

Estimates of the alpha diversity (Observed, Chao1, ACE, Shannon) of microbial communities of soil samples were calculated depending on the type of consortium with which seeds were inoculated with before planting (Supplementary Table S6). Box and whisker diagrams were constructed for comparison (Supplementary Figure S7).

A pairwise comparison of the alpha-diversity indices (observed and Shannon) by the Wilcoxon test did not show a significant difference between soil samples from seeds inoculated with different consortia.

**Figure 5:**
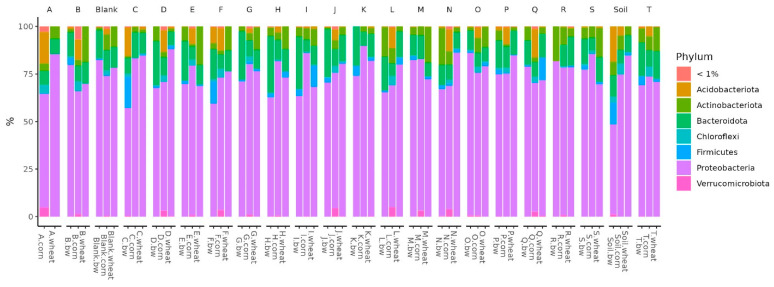
Relative abundance histograms of dominant bacteria phyla in each soil sample. The letters on figure are consortia codes.

In all the studied soil samples, the dominant bacterial phyla were Proteobacteria, Firmicutes, Bacteroidota, Actinobacteroidota, and Acidobactoroidota. It is noteworthy that the proportion of Firmicutes increased in the three soil samples. All these samples belonged to plots with buckwheat, two had plants whose seeds were inoculated by Consortia C and F, and the third was a control plot of soil on which plants were not grown (Figure 5).

**Figure 6:**
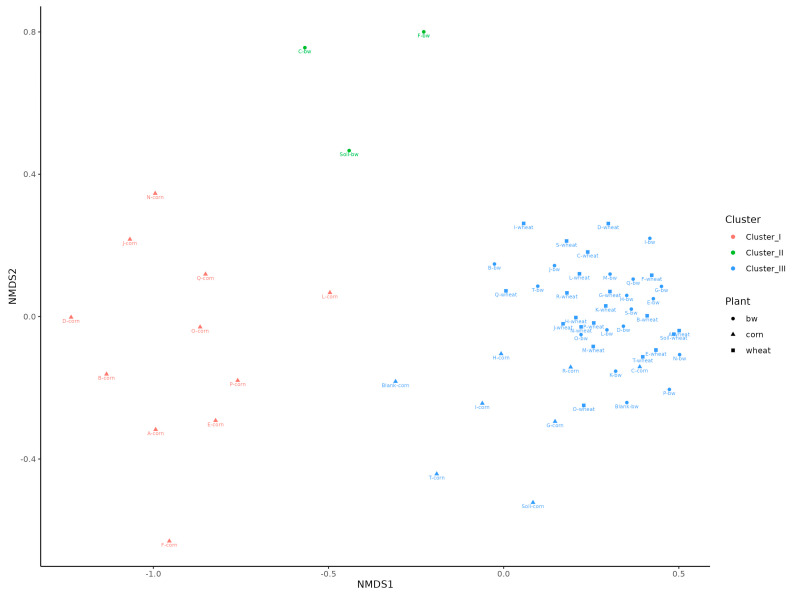
A non-metric multidimensional scaling (NMDS) of soil bacterial community composition among testing samples.

A beta diversity analysis using non-metric multidimensional scaling (NMDS) showed that the samples were clearly divided into three clusters. The first cluster predominantly included samples from corn plots, the second from the C, F and Soil from buckwheat plots, and the third the remaining samples (Figure 6).

**Figure 7:**
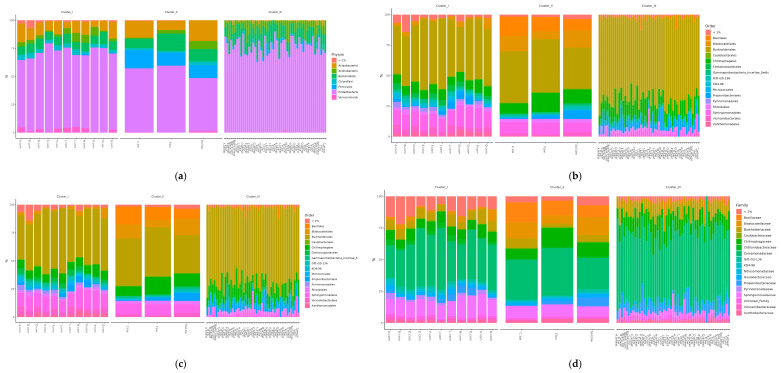
Relative abundance histograms of dominant bacteria phyla (**a**), class (**b**), order (**c**), and family (**d**) in each soil sample grouped by clusters from NMDS.

To assess the differences in the representation of different bacteria between the clusters defined by the analysis, several additional relative abundance histograms were built (Figure 7a–d).

Histograms show the difference in the representation of bacteria between clusters at different levels of classification: phylum, class, order, and family. At the phylum level in the samples of Cluster 2, the amount of proteobacteria was significantly reduced, while firmicutes and acidobacteriota were increased. At the class level in Cluster 2, the content of bacilli was increased, and gammaproteobacteria was reduced. At the level of orders in class 2, bacillales was elevated, and at the level of the family bacillaceae was increased.

**Figure 8:**
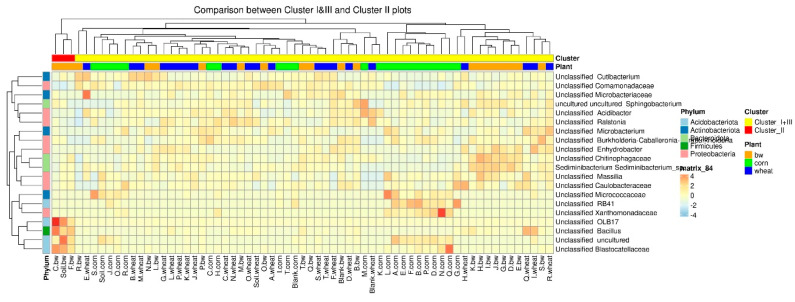
The heatmap of differentially abundant taxa between the samples of cluster II and union of clusters I and III.

To estimate the differential abundance of taxa between the groups, a differential abundance analysis was carried out. Cluster 2, containing a sample of C.bw, and the union of Clusters 1 and 3 were initially taken as comparison groups (Figure 8). In the heat map, the red square marks OTUs, the representation of which is significantly higher in this group compared to the rest. The samples of Cluster II 4 OTUs are more abundant: *Unclassified OBL17*, *Unclassified Bacillus*, *Unclassified Blastocatellaceae*, and *Unclassified Uncultured*.

**Figure 9:**
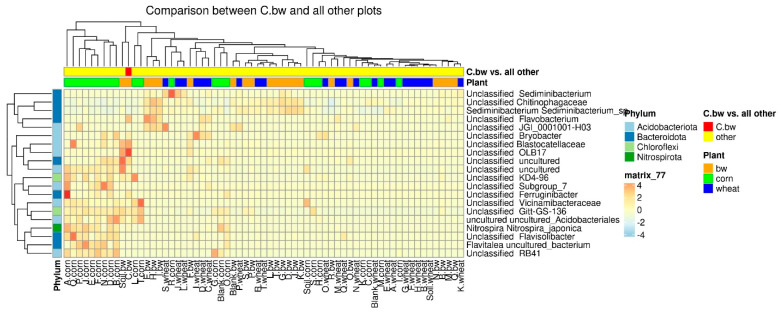
The heatmap of differentially abundant taxa between the C.bw sample and all other samples.

**Figure 10:**
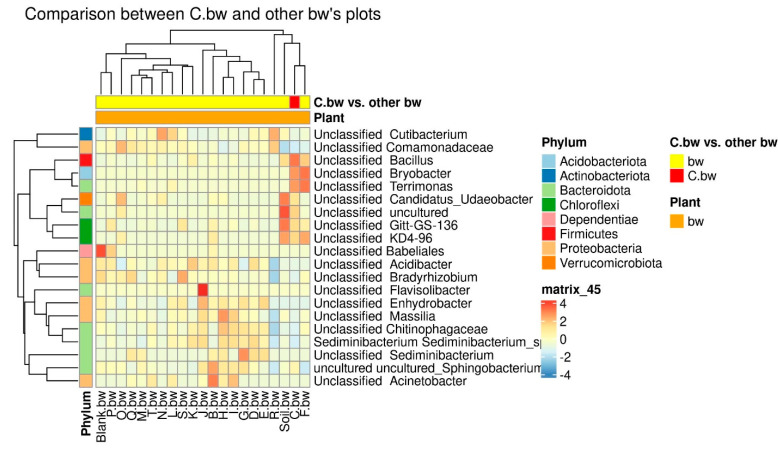
The heatmap of differentially abundant taxa between the C.bw sample and all other buckwheat samples.

Due to the fact that the plants and the composition of the soil from the C.bw plot showed the highest agrotechnical indicators in this experiment, two comparisons were carried out: C.bw against all other samples (Figure 9) and C.bw against all samples from plots with buckwheat (Figure 10).

The results of the comparison confirmed the previously identified *Unclassified OBL17* and *Unclassified Bacillus*.

The plot C.bw was distinguished from all other plots (except for F.bw) with buckwheat by *Unclassified Bacillus (Firmicutes)*, *Unclassified Bryobacter (Acidobacteriota)*, *Unclassified Terrimonas (Bacteroidota).* Also, the content of Unclassified Candidatus_Udaeobacter, Unclassified Gitt-GS-136, Unclassified KD4-96, and Unclassified uncultured was increased. It is noteworthy that an increase in the content of KD4-96 was detected for all three samples included in Cluster II (C.bw, F.bw, and Soil.bw).

**Figure 11:**
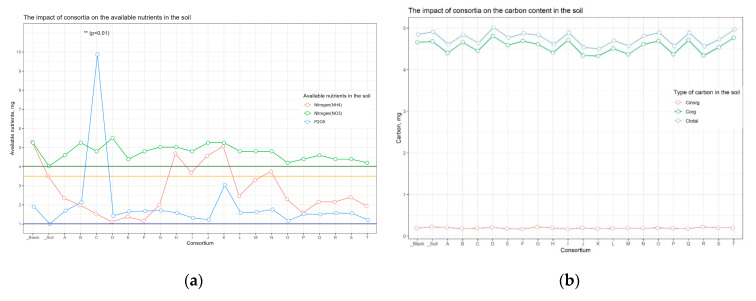
The available soil nutrients and carbon content on the buckwheat plots’ (**a**) nutrients, and (**b**) carbon content.

Since Cluster 2 contained only samples from plots allocated for buckwheat, the available soil nutrients and carbon content were only separately compared between buckwheat plots, in order to neutralize the influence of different crops on soil nutrients (Figure 11). With the exception of the increased content of available phosphorus in plot C.bw (*p* < 0.01), the content of other nutrients did not statistically significantly differ between plots.

**Figure 12:**
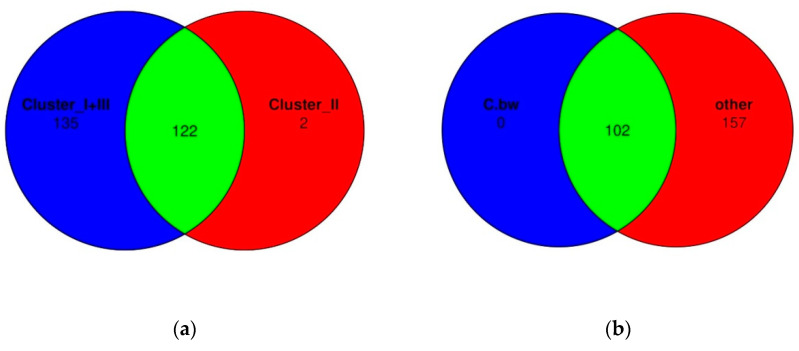
Comparison of bacterial OTU between clusters using Venn diagrams. (**a**) Cluster II vs. Clusters I and III; (**b**) C.bw vs. other samples.

Finally, Venn diagrams were constructed in order to assess whether there were unique bacterial taxa in Cluster II or in the soil of the plot from C.bw (Figure 12).

According to Venn diagrams, Cluster 2 had two unique OTUs. Both OTUs are defined at the family level, moreover, both belong to the same family: domain *Bacteria*, phylum *Acidobacteriota*, class *Blastocatellia*, order *Blastocatellales*, family *Blastocatellaceae.* Both OTUs are not unique to C.bw; they were identified in two samples: C.bw and Soil.bw. Since the Soil.bw sample represents the soil microbiome before the experiment, it can be argued that these OTUs were likely present initially and were only preserved in plot C.bw. Other taxa, which exhibited increased content in the soil sample of the C.bw plot, were likely present in the soil before planting. However, under the influence of external factors, these taxa gained a selective advantage in reproduction.

## 3. Discussion

The inoculation of seeds from important agricultural crops (wheat, buckwheat, corn) with bacterial consortia, followed by cultivation in the open field, improved the agrotechnical indicators for buckwheat treated by Consortium C. While for other variants of the culture–consortium interaction, the agrotechnical indicators either did not differ from the control or exhibited variations in just one indicator, for the combination “Consortium C—buckwheat” there was a systemic positive improvement in all growth and yield indicators.

Consortium C was based on the Rothia endophytica GMG9 strain, which demonstrated a high phosphate-immobilizing ability (248.3 µg/mL) in a laboratory experiment [13]. An active nitrogen fixator, the strain Azotobacter chroococcum GMG39 [13], was also added to Consortium C. Interestingly, another Consortium H, created on the basis of the Rothia endophytica GMG9 strain, but with the addition of a strain with high siderophore production and antifungicidal activity (Enterobacter amnigenus GMG288 [13]), did not demonstrate an increase in available phosphates in the soil. Also, Consortia B, D, and E, which contained Azotobacter chroococcum GMG39 in combination with other phosphate-immobilizing bacteria, did not show any effect on the availability of phosphates in the soil and buckwheat yield. Thus, it can be assumed that, in Consortium C, one can observe a synergistic effect of the strains Rothia endophytica GMG9 and Azotobacter chroococcum GMG39. The synergistic effects of the use of nitrogen-fixing and phosphate-immobilizing bacteria strains have already been demonstrated, on both the macronutrient contents in plants and their availability in the soil [6,7]. In most cases, phosphate mobilizers improve nitrogen fixation by increasing the availability of phosphates for the operation of nitrogenases or the development of the root system [14]. At the same time, it is worth noting that this synergistic effect was present only when growing buckwheat and did not manifest itself in any way when growing wheat or when growing corn. This can be explained by the fact that plants have a strong influence on the composition of microbial communities in the soil through the release of root exudates and decomposition of litter and roots. The relationship between plant species and microbial communities in rhizospheric soil is strict and is the result of co-evolution [15]. Initially, the species Rothia endophytica was described as isolated from superficially sterilized roots of Dysophylla stellata (Lour.)—a plant of the family of Lamiaceae, used as a medicinal substance in China [16]. Perhaps this bacterium can only interact with the roots of some plant species, one can see the effect of the successful symbiosis of bacteria and plants. Unfortunately, plant root samples were not preserved in the work, and this issue requires further research.

Analyzing microbial diversity in the soils of experimental samples three months after inoculation, no strains that formed consortia were found. There are examples of both the long-term presence of inoculants in the rhizosphere of plants [17] and their temporary effect for up to three days [18]. This suggests that when seeds are inoculated, the main effects are as follows: (1) the initial effect of bacteria embedded in the seed peel on the seedling; (2) modification of the habitat and subsequent changes in the composition of the soil microbiome. Hypothesis 1 does not explain the serious change in soil composition that occurred three months after sowing seeds treated by the consortium, so it is assumed that hypothesis 2 is the most likely.

The formation of a soil microbial community is a complex process that is determined by many factors, including the physico-chemical characteristics of the soil, vegetation, and random events, which leads to the formation of a stable community where all functional niches are occupied [19]. In this regard, an increase in alpha diversity is often associated with better soil productivity, since a variety of microorganisms provide nutrition and protect the plant from stress in different conditions [20]. However, it was shown that a decrease in the alpha diversity of the soil before the inoculation of Azospirillum brasilense reduced interspecific microbial competition and triggered the processes of the recolonization of plants by bacteria associated with PGPB effects [21]. The species richness of the soil microbial community (alpha diversity) was similar for all studied samples. That is, the effect of the introduction of consortia did not lead to an increase in the wealth of the microbial community in our case. However, it is more important to assess the ratio of different phylogenetic groups of microorganisms [22]. Beta-diversity analysis revealed that the microbial diversity of three soil samples (C.bw, F.bw and Soil.bw) differed significantly from all others and these samples were allocated to a separate cluster by NMDS in Cluster II. At the phylum level, the number of Acidobacteriota and Firmicutes in samples from Cluster II was increased. This is consistent with the visualization of the results comparing the union of Clusters I and III with Cluster II on the heatmap. Cluster II samples contain an increased amount of OTUs: Unclassified OBL17 (Acidobacteriota), Unclassified Blastocatellaceae (Acidobacteriota) and Unclassified uncultured (Acidobacteriota) and Unclassified Bacillus (Firmicutes). Moreover, according to the Venn diagram, Cluster II has two unique OTUs, both of which belong to the phylum Acidobacteriota, class Blastocatellia, order Blastocatellales, and family Blastocatellaceae.

Due to the difficulties of cultivation and laboratory maintenance, many classes of Acidobacteriota contain a limited number of well-characterized representatives. Due to approaches to the identification of bacteria that exclude cultivation, new classes of Acidobacteriota, called subdivisions (SDs), have been identified. Unclassified OBL17 (Acidobacteriota) [23] and Unclassified Blastocatellaceae (Acidobacteriota) [24], which distinguish Cluster II from the union of Clusters I and III in this study, belong to the SD4 subtype. Lauber et al. revealed a clear correlation of the representation of Acidobacteria depending on the pH of the soil [25]. Hartman et al. observed a strong increase in the abundance of Acidobacteria with a lower pH. Their results reveal shifts in the composition of whole bacterial communities and the abundance of specific taxonomic groups with environmental gradients that may reflect changes in biogeochemical cycling [26]. Despite there being no published data on soil acidity for Unclassified OLB17 (Acidobacteriota) and Unclassified Blastocatellaceae (Acidobacteriota), the fact that a very high value of available phosphorus was recorded for soil sample C.bw indirectly indicates that the pH of this sample was acidic.

It is noteworthy that the sample F.bw also fell into Cluster II, but the plants from this plot did not show such high agrotechnical indicators, and the soil did not have a high content of available phosphorus. C.bw and Soil.bw, as opposed to F.bw, included unique OTUs belonging to the Blastocatellaceae family. These bacteria are known as oligotrophic [27], demonstrate tolerance to a wide pH and temperature range [28], and can participate in soil bioremediation [29].

It is possible that this genus of bacteria, present in the original soil, is suppressed when planting cultivated plants, since they were not found in any other samples. At the same time, the introduction of consortium C into buckwheat preserved the conditions for the favorable existence of the family Blastocatellaceae. Perhaps this factor is the acidic pH of the soil, since, in laboratory studies, the Rothia endophytica GMG9 strain demonstrated a high ability to immobilize phosphates, which, in turn, is often due to acid production. Unfortunately, the family Blastocatellaceae is mainly represented by uncultivated bacteria, and it is difficult to delineate their functions in the soil. Based on the available data, it can be assumed that representatives of this family can participate in the elimination of some substances from the soil that inhibit plant growth or the accompanying beneficial microflora. Considering this scenario, the bacteria introduced through inoculation might have a more enduring effect in such an environment.

It is interesting to note that the soil microbiome following the introduction of consortium C aligned with a soil sample taken from a plot where no crops were grown. This suggests that the addition of Consortium C potentially conserved the original microbial community of the soil. At the same time, the buckwheat harvest was higher than that in all other plots. It can be assumed that the planted plant secretes some substances into the soil that modify the microbial community, adjusting it to the needs of the plant, but suppressing the growth of some important bacteria that help enrich the soil with nutrients. In the case of consortium C, the introduced bacteria either neutralized this action or “protected” the family Blastocatellaceae bacteria from it, which allowed them (or some other bacteria) to survive and have a further beneficial effect on the plant. This may also explain the specific manifestation of consortium C’s effect on buckwheat, since different plants most likely possess their own array of such metabolites.

Therefore, this study showed that, under the conditions of natural gray forest soils, the introduction of a consortia of bacteria beneficial to plants did not have a significant effect on the growth and yield of wheat, corn, and buckwheat, with the exception of consortium C for buckwheat. The only successful result (consortium C for buckwheat) showed that, when selecting microorganisms for the creation of microbial fertilizers, it is important to take into account not only the characteristics of the soil but also the characteristics of the plant, which can affect the survival of beneficial bacteria in the rhizosphere. It was also shown that such a favorable result was associated with a significant increase in the availability of phosphates in the soil, which can be attributed both to the direct action of a consortium of phosphate-immobilizing and nitrogen-fixing bacteria and to the acidification of the medium due to an increase in phylum Acidobacteriota bacteria in the soil. An analysis of the soil microbiota after the introduction of consortia showed that the addition of consortium C during buckwheat cultivation led to the preservation of the original soil microbiome. This preservation might be the cause of the high yield in this plot, suggesting that this could be another mechanism by which beneficial bacteria contribute to plant growth.

## 4. Materials and Methods

### 4.1. Designing Bacterial Consortia

Twenty consortia of 2–3 bacteria were used for the study (Table 1). For ease of citation, these 20 consortia are coded with letters of the Latin alphabet from A to T. All consortia included microorganisms from the collection (Center of Applied Microbiology of the Institute of Chemical Biology and Fundamental Medicine (ICBFM), Novosibirsk, Russia) that showed the best results in tests of phosphate solubilization or the ability to grow in a nitrogen-free environment and ammonia production. Seven consortia were based on *Pseudomonas koreensis GMG11* strain with high phosphate-immobilizing capacity (294.8 µg/mL); six of them also included various strains with a high ability to grow on a nitrogen-free medium (A, D, M, P, S, T); and one strain had a high production of auxin (Q). Another 6 consortia (K, B, I, L, O, R) were based on the *Rahnella aceris GMG294* strain with a high phosphate-immobilizing ability (214.0 µg/mL), to which different strains with a high ability to grow on a nitrogen-free medium were also added. Also, four consortia were based on Pseudomonas strains that demonstrated high siderophore production values (F, G, E, N) with the addition of either phosphate-immobilizing (F, G) or nitrogen-fixing (E, N) bacteria. All the consortia described above are composed of bacteria belonging to the Pseudomonadota phylum; three more consortia were composed based on strains from another phylum—Actinomycetota (C,H,J). Consortia C and H were based on *Rothia endophytica GMG9* with a high phosphate-immobilizing ability (248.3 µg/mL), with the addition of nitrogen-fixing bacterium *Azotobacter chroococcum GMG39* (C) or a strain with high antifungicidal activity *Enterobacter amnigenus GMG288* (H). Consortium J was based on the nitrogen-fixing strain of *Rhodococcus erythropolis GMG21* with the addition of the phosphate-immobilizing strain *Pseudomonas koreensis GMG11*. When composing combinations, preliminary experiments were carried out on the antagonistic activity of strains so that growth suppression did not occur.

As comparison samples, we added two more plots for each crop: Blank and Soil. Crops were grown on the Blank plot, the seeds of which were inoculated with sterile water instead of a bacterial consortium before planting. This was a control for the effect of inoculation by the consortium. No crops were planted on the Soil plot, which was a control for the soil’s condition before planting cultivated plants.

### 4.2. Description of the Field Experiment

The effect of bacterial inoculation on plant growth was studied on three significant crops: corn, buckwheat, and wheat. All the selected seeds were surface-sterilized by 1% NaOCl for 90 s and two consecutive rinses in sterile distilled water, followed by air-drying under laminar air flow. Bacterial cultures were grown in 50 mL falcon tubes filled with 10 mL LB broth and were kept on shaker at 200 rpm for 48 h and diluted to adjust 108 cfu/mL bacterial solutions with sterile distilled water. Seeds were coated with culture by immersion in a suspension of bacteria for 120 min. This experiment was carried out in three replications and the results were compared with control seeds treated with water instead of a bacterial isolate. Five seeds were placed in each plot at a depth of 2–3 cm. The experiment lasted 90 days, until the grain matured. The experiment was set up in a randomized design, with three biological replications.

The soils on which small-scale field experiments were carried out belong to the type of gray forest soils. Within the Novosibirsk region, gray forest soils are found in the forest-steppe zone, the taiga subzone and, much less often, in the southern taiga subzone. In the right-bank part of the Siberian district (the Salair drained plain, the foothills of the Salair ridge), gray forest soils occupy about 1250 thousand hectares in such administrative districts as Novosibirsk, Maslyaninsky and Iskitimsky, which is more than 50% of all land. In the left-bank part, gray forest soils are much less common and occupy about 50 thousand hectares, mainly in the northern part of the Priobsky plateau and in places north of the Barabinsk and south of the Privasyugan lowland plains (Kolyvan, Mikhailovsky, Severny, Kyshtovsky administrative districts). The total area of gray forest soils in the region is about 1.3 million hectares (7.8% of all land). Gray forest soils are formed on elevated landforms with an abundence of surface runoff and relatively deep groundwater levels. Natural vegetation is mainly represented by small-leaved birch and birch–aspen forests and meadow steppes.

### 4.3. Determination of Chemical Parameters of the Soil

Soil samples were analyzed for the content of available phosphorus (AP), exchangeable potassium (Ex-K), available nitrogen (AN-NO_3_, AN-NH_4_), soil organic (SOC) and soil inorganic carbon (SIC) with different protocols [30]:

(1) Soil organic carbon—0.1–0.2 g soil, reaction with 0.4 N K_2_Cr_2_O_7_ in mixture with H_2_SO_4_;

(2) Available phosphorus—20 g soil extracted by 0.03 N K_2_SO_4_, 5 min reaction time;

(3) Exchangeable potassium—5 g soil < 1.0 mm, extracted by 50 mL of CH_3_COONH_4_, pH = 7, 1 h reaction time.

The labile forms of macronutrients (AN-NO_3_, AN-NH_4_) were determined by the conservative methods described by Maynard and coauthors [31]. In brief, the quantity of nitrate was determined potentiometrically after the extraction of 2 g of the dry cadaver material by 20 mL of 0.03M K_2_SO_4_. Ammonium content was determined colorimetrically after extraction of 2.5 g of the cadaver material by 50 mL of the 2N KCl. Each treatment was replicated three times. 

The content of soil organic (SOC) and soil inorganic carbon (SIC) were determined by stepwise loss on ignition method using 2–4 g soil aliquots [32].

### 4.4. Determination of the Yield of Buckwheat, Wheat and Corn

To assess the yield of crops grown as part of the experiment, the following parameters were recorded: plant height, mass of dry straw, mass of grain from the plot, mass of dry roots. The height of the plants was measured after 2, 4 and 6 weeks. The mass of dry roots, the mass of straw and the mass of grain were measured three months after harvest. For grain, the average weight from three plots was estimated (mean and SD), as well as the average amount of grain per plot, taking into account fallen grain calculated as the sum of the weight of grain from three plots and fallen grain divided by three.

### 4.5. Soil DNA Extraction and NGS-Sequencing

#### 4.5.1. DNA Extraction

Total DNA from 0.5g soil was extracted using MagBeads FastDNA^TM^ Kit for Soil (MP Biomedicals, Irvine, CA, USA), as recommended by the manufacturer. DNA quantity was estimated by Qubit 4.0 (Invitrogen/Life Technologies, Carlsbad, CA, USA).

#### 4.5.2. Sequencing of 16S rRNA Gene Libraries

The purified DNA isolates were amplified with primers previously developed by us, Artik-FF (5′- GTGACTGGAGTTCAGACGTGTGCTCTTCCGATCTCTACGGGAGGCAGCAG-3′) and (Artik-FR 5′-ACTCTTTCCCTACACGACGCTCTTCCGATCTGGACTACCGGGGTATCT-3′), targeting variable regions V3–V4 of bacterial and archaeal 16S rRNA genes. PCR was carried out in a 25 μL reaction mixture containing 1 unit of Q5 Hot Start High-Fidelity DNA Polymerase and Q5 Reaction Buffer (New England Biolabs, Ipswich, MA, USA), 10 pM of each primer, 2 ng of DNA matrix and 2 nM of each dNTP and fluorescent dye SYBR Green. Amplification was performed in a CFX96 (Bio-Rad, Hercules, CA, USA) under the following conditions: initial denaturation for 3 min at 96 °C, then 40 cycles consisting of denaturation at 95 °C for 10 s, followed by the annealing of primers at 55 °C for 30 s and subsequent elongation at 72 °C for 30 s The final elongation was carried out at 72 °C for 5 min. Visualization of PCR products was carried out by gel electrophoresis in agarose gels in the presence of ethidium bromide. PCR products were purified according to the recommended Illumina technique using AM Pure XP (Beckman Coulter Life Sciences, Indianapolis, IN, USA). DNA concentration in solutions was determined using a desktop fluorimeter Qubit 4.0 (Invitrogen/Life Technologies USA). To achieve this, we used the Qubit dsDNA HS Assay Kit according to the protocol. Enrichment was carried out using PCR. A set of oligonucleotides Multiplex Oligos for Illumina Dual Index Primer Set 1 (New England Biolabs, USA) was used as primers. PCR was carried out in a 25 μL reaction mixture containing 1 units Q5 Hot Start High-Fidelity DNA Polymerase and Q5 Reaction Buffer (New England Biolabs, USA), 10 pM of each primer, DNA matrix and 2 nM of each dNTP and fluorescent dye SYBR Green. Amplification was performed in an CFX96 (Bio-Rad, USA) under the following conditions: initial denaturation for 30 s at 98 °C, then 12 cycles consisting of denaturation at 98 °C for 10 s, followed by the annealing of primers and elongation at 65 °C for 75 s. The final elongation was carried out at 65 °C for 5 min. Then, PCR products were purified according to the recommended Illumina technique using AM Pure XP (Beckman Coulter Life Sciences, USA). To create a single pool of all libraries, we calculated how much of each library should be taken to obtain the same amount of DNA in nanograms. Calculations were made based on the concentrations of DNA in the enriched libraries measured using the Qubit 4.0 desktop fluorimeter. To quantify amplicon libraries, the Real-Time PCR method was used with the addition of a TaqMan probe with a ROX dye label (Syntol, Moscow, Russia) and oligonucleotides complementary to the end-sequences of libraries. As a quantitative standard, PhiX-control was used, diluted 10, 100, 1000 and 10,000 times. The concentration of the library pool was determined by the location of the fluorescence signal accumulation curve relative to the control samples. The 16S rRNA gene amplicons were sequenced in paired-end mode (2 × 301) with the Illumina MiSeq v3 (600 cycles) kit (Illumina, San Diego, CA, USA) on a MiSeq at Sirius University of Science and Technology (Sirius, Russia).

### 4.6. Statistical Analysis

The yield of plants is expressed in terms of the mean and SD. Visualization was achieved using function ggplot () in R. 

Analysis of 16S ribosomal RNA gene sequences was performed using QIIME 2 v.2023.7 (https://docs.qiime2.org/2023.9/install/, (accessed on 23 August 2023)) [33]. Sequence quality control, merging of paired-end reads and chimera filtering were carried out with Deblur plugin [34]. Operational Taxonomic Units (OTUs) were clustered by applying VSEARCH plugin [35] with cluster-features-closed-reference using dbSilva 138 SSURef NR99 [36] with 97% identity. The taxonomy assignment was performed using global search alignment (feature-classifier classify-sklearn). Since, according to the recommendations, the accuracy of the taxonomic classification of 16S rRNA gene sequences increases when the naive Bayesian classifier is trained only on that area of the target sequences, which was sequenced, as a classifier, we used our own trained naïve Bayesian classifier, obtained using the SILVA database reference sequences, which were limited to the primer sequences used for sequencing, as described i nthe following (https://docs.qiime2.org/2023.9/tutorials/feature-classifier/, (accessed on 23 August 2023)). To generate a rooted phylogenetic tree, the align-to-tree-mafft-fasttree pipeline from the q2-phylogeny pluginwas used. The resolution curves were constructed using the diversity alpha-rarefaction plugin.

The description and visualization of alpha and beta diversity was performed using the following packages in R: phyloseq [37], DESeq2 [38], ComplexHeatmap [39].

## Figures and Tables

**Table 1:** Bacterial strains included in the formed consortia.

Consortium Code	A Strain with a High Ability to Grow in a Nitrogen-Free Environment	Strains with a High Ability to Solubilize Phosphates	Strain with a High Production of Auxin	Strain with a High Production of Siderophore or Antifungal Activity
based on *Pseudomonas koreensis GMG11*				
A		Pseudomonas koreensis GMG11		
D	Azotobacter chroococcum GMG39	Pseudomonas koreensis GMG11		
M	Enterobacter cloacae GMG24	Pseudomonas koreensis GMG11		
P	Rahnella aquatilis GMG287	Pseudomonas koreensis GMG11		
S	Hylemonella gracilis GMG31	Pseudomonas koreensis GMG11		
T	Agrobacterium arsenijevicii GMG33	Pseudomonas koreensis GMG11		
Q		Pseudomonas koreensis GMG11	Enterobacter ludwigii GMG278	
based on the *Rahnella aceris GMG294*				
K		Rahnella aceris GMG294		
B	Azotobacter chroococcum GMG39	Rahnella aceris GMG294		
I	Variovorax paradoxus	Rahnella aceris GMG294		
L	Enterobacter cloacae GMG24	Rahnella aceris GMG294		
O	Hylemonella gracilis GMG31	Rahnella aceris GMG294		
R	Pantoea agglomerans GMG20	Rahnella aceris GMG294		
based on strains with “antibiotic” qualities				
E	Azotobacter chroococcum GMG39			Pseudomonas kitaguniensis GMG234
N	Enterobacter cloacae GMG24			Pseudomonas kitaguniensis GMG234
F		Pseudomonas kitaguniensis GMG14		Pseudomonas silesiensis GMG271
G		Pseudomonas kitaguniensis GMG14		Pseudomonas kitaguniensis GMG219
based on strains Pseudomonadota and Actinomycetota				
C	Azotobacter chroococcum GMG39	Rothia endophytica GMG9		
H		Rothia endophytica GMG9		Enterobacter amnigenus GMG288
J	Rhodococcus erythropolis GMG21	Pseudomonas koreensis GMG11		

## Data Availability

Data are contained within the article and Supplementary Materials.

## Supplementary Materials

The following supporting information can be downloaded at: https://www.mdpi.com/article/10.3390/plants13010116/s1, Figure S1: Wheat’s height on 4 and 6 weeks; Figure S2: Wheat’s length of ear and steam of after 3 months; Figure S3: Weight of wheat’s ear and grain; Figure S4: Corn’s height on 4 and 6 weeks; Figure S5: Weight of corn’s ears and roots; Figure S6: Dependence of detection of the number of taxa (OTUs) on the number of sequences (N); Figure S7: Box plot of indices of alpha diversity of microorganisms in soil plots; Table S1: Buckwheat’s yield parameters of height; Table S2: Buckwheat’s yield parameters estimated by weight of grain, straw and roots; Table S3: Wheat’s yield parameters of height; Table S4: Corn’s yield parameters; Table S5: Soil nutrient status; Table S6: Statistical table of α-diversity index under different consortium on wheat, corn and buckwheat.

## References

[1] Richardson A.E., Barea J.-M., McNeill A.M., Prigent-Combaret C. (2009). Acquisition of phosphorus and nitrogen in the rhizosphere and plant growth promotion by microorganisms. Plant Soil.

[2] Jeon J.S., Lee S.S., Kim H.Y., Ahn T.S.S.H. (2003). Plant Growth Promotion in Soil by Some Inoculated Microorganisms. J. Microbiol..

[3] Timmusk S., Nicander B., Granhall U., Tillberg E. (1999). Cytokinin production by *Paenibacillus polymyxa*. Soil Biol. Biochem..

[4] Gutie’rrez-Mañero F.J., Ramos-Solano B., Probanza A., Mehouachi J., Tadeo F.R., Talon M. (2001). The plant-growth-promoting rhizobacteria *Bacillus pumilus* and *Bacillus licheniformis* produce high amounts of physiologi-cally active gibberellins. Physiol. Plant..

[5] Kloepper J.W., Leong J., Teintze M., Schroth M.N. (1980). Enhanced plant growth by siderophores produced by plant growth-promoting rhizobacteria. Nature.

[6] Yu X., Liu X., Zhu T.-H., Liu G.-H., Mao C. (2012). Co-inoculation with phosphate-solubilzing and nitrogen-fixing bacteria on solubilization of rock phosphate and their effect on growth promotion and nutrient uptake by walnut. Eur. J. Soil Biol..

[7] Wani P.A., Khan M.S., Zaidi A. (2007). Synergistic effects of the inoculation with nitrogen-fixing and phosphate-solubilizing rhizobacteria on the performance of field-grown chickpea. J. Plant Nutr. Soil Sci..

[8] Zhan Y., Xu S., Hou Z., Gao X., Su J., Peng B., Zhao J., Wang Z., Cheng M., Zhang A. (2023). Co-inoculation of phosphate-solubilizing bacteria and phosphate accumulating bacteria in phosphorus-enriched composting regulates phosphorus transformation by facilitating polyphosphate formation. Bioresour. Technol..

[9] JJabborova D., Kannepalli A., Davranov K., Narimanov A., Enakiev Y., Syed A., Elgorban A.M., Bahkali A.H., Wirth S., Sayyed R.Z. (2021). Co-inoculation of rhizobacteria promotes growth, yield, and nutrient contents in soybean and improves soil enzymes and nutrients under drought conditions. Sci. Rep..

[10] Wang Z., Chen Z., Fu X. (2019). Integrated Effects of Co-Inoculation with Phosphate-Solubilizing Bacteria and N2-Fixing Bacteria on Microbial Population and Soil Amendment Under C Deficiency. Int. J. Environ. Res. Public Health.

[11] Kundu B.S., Gaur A.C. (1980). Establishment of nitrogen-fixing and phosphate-solubilising bacteria in rhizosphere and their effect on yield and nutrient uptake of wheat crop. Plant Soil.

[12] Belimov A.A., Kojemiakov A.P., Chuvarliyeva C.N. (1995). Interaction between barley and mixed cultures of nitrogen fixing and phosphate-solubilizing bacteria. Plant Soil..

[13] Voronina E., Sokolova E., Tromenschleger I., Mishukova O., Hlistun I., Miroshnik M., Savenkov O., Buyanova M., Ivanov I., Galyamova M. (2023). Properties of Potential Plant-Growth-Promoting Bacteria and Their Effect on Wheat Growth Promotion (*Triticum aestivum*) and Soil Characteristics. Microbiol. Res..

[14] Janati W., Benmrid B., Elhaissoufi W., Zeroual Y., Nasielski J., Bargaz A. (2021). Will Phosphate Bio-Solubilization Stimulate Biological Nitrogen Fixation in Grain Legumes?. Front. Agron..

[15] Nannipieri P., Ascher J., Ceccherini M.T., Landi L., Pietramellara G., Renella G. (2003). Microbial diversity and soil functions. Eur. J. Soil Sci..

[16] Xiong Z.-J., Zhang J.-L., Zhang D.-F., Zhou Z.-L., Liu M.-J., Zhu W.-Y., Zhao L.-X., Xu L.-H., Li W.-J. (2013). *Rothia endophytica* sp. nov., an actinobacterium isolated from Dysophylla stellata (Lour.) Benth. Int. J. Syst. Evol. Microbiol..

[17] Wang F., Wei Y., Yan T., Wang C., Chao Y., Jia M., An L., Sheng H. (2022). *Sphingomonas* sp. Hbc-6 alters physiological metabolism and recruits beneficial rhizosphere bacteria to improve plant growth and drought tolerance. Front. Plant Sci..

[18] Qiao J., Yu X., Liang X., Liu Y., Borriss R., Liu Y. (2017). Addition of plant-growth-promoting *Bacillus subtilis* PTS-394 on tomato rhizosphere has no durable impact on composition of root microbiome. BMC Microbiol..

[19] Carlström C.I., Field C.M., Bortfeld-Miller M., Müller B., Sunagawa S., Vorholt J.A. (2019). Synthetic microbiota reveal priority effects and keystone strains in the *Arabidopsis phyllosphere*. Nat. Ecol. Evol..

[20] Chen Q.-L., Ding J., Zhu Y.-G., He J.-Z., Hu H.-W. (2020). Soil bacterial taxonomic diversity is critical to maintaining the plant productivity. Environ. Int..

[21] Ferrarezi J.A., Defant H., de Souza L.F., Azevedo J.L., Hungria M., Quecine M.C. (2023). Meta-omics integration approach reveals the effect of soil native microbiome diversity in the performance of inoculant *Azospirillum brasilense*. Front. Plant Sci..

[22] Trabelsi D., Mengoni A., Ben Ammar H., Mhamdi R. (2011). Effect of on-field inoculation of Phaseolus vulgaris with rhizobia on soil bacterial communities. FEMS Microbiol. Ecol..

[23] Damsté J.S.S., Rijpstra W.I.C., Foesel B.U., Huber K.J., Overmann J., Nakagawa S., Kim J.J., Dunfield P.F., Dedysh S.N., Villanueva L. (2018). An overview of the occurrence of ether- and ester-linked iso-diabolic membrane lipids in microbial cultures of the Acidobacteria: Implications for brGDGT paleoproxies for temperature, pH. Org. Geochem..

[24] Dedysh S.N., Sinninghe Damsté J.S. (2018). Acidobacteria.

[25] Lauber C.L., Hamady M., Knight R., Fierer N. (2009). Pyrosequencing-Based Assessment of Soil pH as a Predictor of Soil Bacterial Community Structure at the Continental Scale. Appl. Environ. Microbiol..

[26] Hartman W.H., Richardson C.J., Vilgalys R., Bruland G.L. (2008). Environmental and anthropogenic controls over bacterial communities in wetland soils. Proc. Natl. Acad. Sci. USA.

[27] Ivanova A.A., Zhelezova A.D., Chernov T.I., Dedysh S.N. (2020). Linking ecology and systematics of acidobacteria: Distinct habitat preferences of the Acidobacteriia and Blastocatellia in tundra soils. PLoS ONE.

[28] Pascual J., Wüst P.K., Geppert A., Foesel B.U., Huber K.J., Overmann J. (2015). Novel isolates double the number of chemotrophic species and allow the first description of higher taxa in *Acidobacteria subdivision* 4. Syst. Appl. Microbiol..

[29] Huber K.J., Geppert A.M., Groß U., Luckner M., Wanner G., Cooper P., Abakah J., Janssen I., Overmann J. (2017). *Aridibacter nitratireducens* sp. nov., a member of the family Blastocatellaceae, class Blastocatellia, isolated from an African soil. Int. J. Syst. Evol. Microbiol..

[30] VSA (1975). Agrokhimicheskiye Metody Issledovaniya Pochv.

[31] Maynard D.G., Kalra Y.P.C.J.A. (2007). Nitrate and exchangeable ammonium nitrogen. Soil Sampling and Methods of Analysis.

[32] Wang Q., Li Y.W.Y. (2011). Optimizing the weight loss-on-ignition methodology to quantify organic and carbonate carbon of sediments from diverse sources. Environ. Monit. Assess. Environ. Monit. Assess..

[33] Caporaso J.G., Kuczynski J., Stombaugh J., Bittinger K., Bushman F.D., Costello E.K., Fierer N., Gonzalez Peña A., Goodrich J.K., Gordon J.I. (2010). QIIME allows analysis of high-throughput community sequencing data. Nat. Methods.

[34] Amir A., McDonald D., Navas-Molina J.A., Kopylova E., Morton J.T., Zech Xu Z., Kightley E.P., Thompson L.R., Hyde E.R., Gonzalez A. (2017). Deblur Rapidly Resolves Single-Nucleotide Community Sequence Patterns. mSystems.

[35] Rognes T., Flouri T., Nichols B., Quince C., Mahé F. (2016). VSEARCH: A versatile open source tool for metagenomics. PeerJ.

[36] Quast C., Pruesse E., Yilmaz P., Gerken J., Schweer T., Yarza P., Peplies J., Glöckner F.O. (2012). The SILVA ribosomal RNA gene database project: Improved data processing and web-based tools. Nucleic Acids Res..

[37] McMurdie P.J., Holmes S. (2013). phyloseq: An R Package for Reproducible Interactive Analysis and Graphics of Microbiome Census Data. PLoS ONE.

[38] McDonald W.I., Ron M.A. (2002). Multiple sclerosis: The disease and its manifestations. Indian J. Pharmacol..

[39] Gu Z., Eils R., Schlesner M. (2016). Complex heatmaps reveal patterns and correlations in multidimensional genomic data. Bioinformatics.

