# Supplementary material for: The Effectiveness of Co-Inoculation by Consortia of Microorganisms Depends on the Type of Plant and the Soil Microbiome
Source: Plants (Basel). 2023 Dec 31;13(1):116. doi: 10.3390/plants13010116 (PMC10780792; doi:10.3390/plants13010116)
Supplement: Supplementary file 1 [file plants-13-00116-s001.zip › plants-2749568-supplementary.pdf]

Supplementary Files

Suppl. Table S1. Buckwheat's yield parameters of height

NS – not significant

Consortium	height of the plants after 2 weeks, cm	height of the plants after 4 weeks, cm	height of the plants after 6 weeks, cm	p-value
A	1,53 ± 0,06	7,72 ± 2,44	51,11 ± 13,21	NS
B	1,40 ± 0,36	7,67 ± 1,61	58,67 ± 9,29	NS
C	1,80 ± 0,28	7,25 ± 0,82	58,00 ± 4,24	0.04
D	1,67 ± 0,29	7,06 ± 1,06	49,56 ± 3,40	NS
E	1,70 ± 0,44	7,67 ± 0,76	52,89 ± 6,11	NS
F	1,63 ± 0,32	8,06 ± 1,42	58,56 ± 4,86	0.04
G	1,45 ± 0,21	8,58 ± 0,12	61,50 ± 4,01	0.02
H	1,10 ± 0,17	6,72 ± 2,25	51,11 ± 3,91	NS
I	1,13 ± 0,23	7,61 ± 1,55	49,22 ± 6,99	NS
J	1,10 ± 0,10	6,06 ± 0,54	52,89 ± 5,70	NS
K	1,33 ± 0,21	7,33 ± 1,01	47,00 ± 3,28	NS
L	1,47 ± 0,21	6,61 ± 2,59	51,67 ± 8,82	NS
M	1,77 ± 0,40	7,94 ± 1,49	49,78 ± 13,04	NS
N	1,70 ± 0,26	7,89 ± 1,50	58,44 ± 8,00	NS
O	1,53 ± 0,47	7,00 ± 2,49	49,22 ± 11,36	NS
P	1,17 ± 0,76	6,89 ± 0,77	49,11 ± 5,00	NS
Q	1,13 ± 0,23	7,89 ± 0,63	48,44 ± 3,85	NS
R	1,17 ± 0,25	6,78 ± 1,54	54,44 ± 2,69	NS
S	1,77 ± 0,59	7,89 ± 1,68	51,44 ± 8,68	NS
T	1,17 ± 0,59	8,22 ± 0,69	55,00 ± 8,09	NS
Blank	1,33 ± 0,29	6,56 ± 0,69	47,00 ± 4,81	NS

Suppl. Table S2. Buckwheat's yield parameters estimated by weight of grain, straw and roots

Consortium	mass of dry straw, g	Mass of grain weight, g (mean ± SE)	average weight of grain from the plot, g	Average weight gain from the plot versus control, %	mass of dry roots, g	Mass of dry roots from the plot versus control, %
A	6,18 ± 1,93	4,70 ± 0,79	7,99	11,4	2,50 ± 0,83	-62,3
B	8,63 ± 1,36	4,11 ± 0,23	6,74	-6,0	10,70 ± 3,57	61,1
C	10,13 ± 5,13	7,27 ± 0,75	10,7	49,2 ($p<0.05$)	16,72 ± 5,57	151,8
D	3,81 ± 0,39	3,07 ± 1,99	5,38	-25,0	4,88 ± 1,63	-26,5
E	5,65 ± 2,49	6,61 ± 3,29	7,64	6,56	5,92 ± 1,97	-10,8
F	6,39 ± 2,66	6,51 ± 4,14	7,88	9,9	8,96 ± 2,99	34,9
G	8,33 ± 3,15	4,12 ± 2,26	8,80	-18,4	7,78 ± 2,59	17,2
H	5,61 ± 0,82	6,43 ± 0,47	7,05	-1,7	8,24 ± 2,75	24,1
I	5,23 ± 2,74	3,47 ± 2,06	5,57	-22,3	6,40 ± 2,13	-3,6
J	4,55 ± 1,25	4,64 ± 2,39	5,53	-22,9	7,44 ± 2,48	12,0
K	5,27 ± 2,04	3,64 ± 0,53	6,26	-12,7	10,02 ± 3,34	50,9
L	6,47 ± 5,32	6,55 ± 5,82	8,05	12,3	6,52 ± 2,17	-1,8
M	6,97 ± 1,90	7,35 ± 3,25	8,77	22,3	9,10 ± 3,03	37,0
N	7,02 ± 1,09	6,22 ± 1,55	7,36	2,6	9,78 ± 3,26	47,3
O	5,27 ± 0,60	4,13 ±1,52	6,23	-13,1	9,92 ± 3,31	49,4
P	3,80 ± 3,32	2,03 ± 1,18	3,12	-56,5 ($p<0.05$)	4,74 ± 1,58	-28,6
Q	5,59 ± 1,40	5,48 ± 1,16	6,06	-15,4	6,20 ± 2,07	-6,6
R	6,47 ± 3,19	4,39 ± 2,00	7,00	-2,3	10,06 ± 3,35	51,5
S	5,98 ± 2,38	6,05 ± 2,45	6,76	-5,7	8,24 ± 2,75	24,1

T	6,43 ± 0,81	3,13 ± 1,64	7,02	-2,1	7,64 ± 2,55	15,1
Blank	6,05 ± 2,55	6,08 ± 2,16	7,17	control	6,64 ± 2,21	control

Suppl. Table S3. Wheat's yield parameters of height

One asterisk (*) marks the level of statistical significance of the differences of 0.05 compared to the control plot (Blank).

Consortium	height of the plants after 4 weeks, cm	height of the plants after 6 weeks, cm	Stem length (straw), cm	Ear length, cm	Stem/ear ratio
A	6,4 ± 1,1	30,3 ± 3,6	67,0 ± 4,8*	4,3 ± 0,4	16
B	6,1 ± 1,0	28,9 ±3,0	52,3 ± 7,6	4,5 ± 0,9	12
C	5,5 ± 0,4	30,6 ± 4,0	53,3 ± 6,5	3,6 ± 0,5	15
D	5,8 ±1,1	30,9 ± 3,0	52,1 ± 7,1	3,5 ± 0,8	15
E	6,8 ± 2,2	34,3 ± 6,3	57,4 ± 6,0	4,5 ± 0,6	13
F	6,3 ± 1,4	29,8 ± 3,9	52,6 ± 6,7	4,6 ± 0,9	11
G	5,2 ± 0,9	29,1 ± 3,5	49,5 ± 5,1	3,3 ± 0,8	15
H	5,4 ± 0,8	30,0 ± 3,1	56,8 ± 5,7	3,4 ± 0,2	17
I	6,2 ± 1,1	32,9 ± 3,1	56,0 ± 6,9	4,1 ± 0,8	14
J	6,4 ± 1,2	31,1 ± 4,8	53,3 ± 3,4	3,3 ± 0,1	16
K	6,2 ± 0,7	34,1 ± 5,0	57,5 ± 7,0	3,9 ± 0,7	15
L	6,6 ±1,5	29,0 ± 4,2	58,4 ± 4,4	4,1 ± 0,4	14
M	6,1 ± 1,0	34,0 ± 3,9	56,8 ± 6,9	4,8 ± 0,5	12
N	6,2 ± 0,5	30,9 ± 6,1	57,1 ± 7,8	3,9 ± 1,2	15
O	5,3 ± 0,4	26,7 ± 3,9	47,7 ± 2,8	3,0 ± 0,6	16
P	7,3 ± 1,5	33,4 ± 2,6	57,4 ± 4,9	3,7 ± 0,2	15
Q	5,8 ± 0,9	31,2 ± 2,3	63,2 ± 5,7	3,8 ± 0,8	17
R	5,9 ± 1,6	29,1 ± 4,4	48,7 ± 2,8	4,1 ± 0,4	12
S	6,3 ± 1,7	32,4 ± 4,3	57,5 ± 8,6	4,1 ± 0,3	14
T	6,1 ± 0,6	32,3 ± 6,0	52,0 ± 2,7	3,8 ± 0,4	14
Blank	5,6 ± 0,5	30,4 ± 2,5	55,1 ± 5,1	3,8 ± 0,8	15

Suppl. Figure S1. Wheat's height on 4 and 6 weeks.

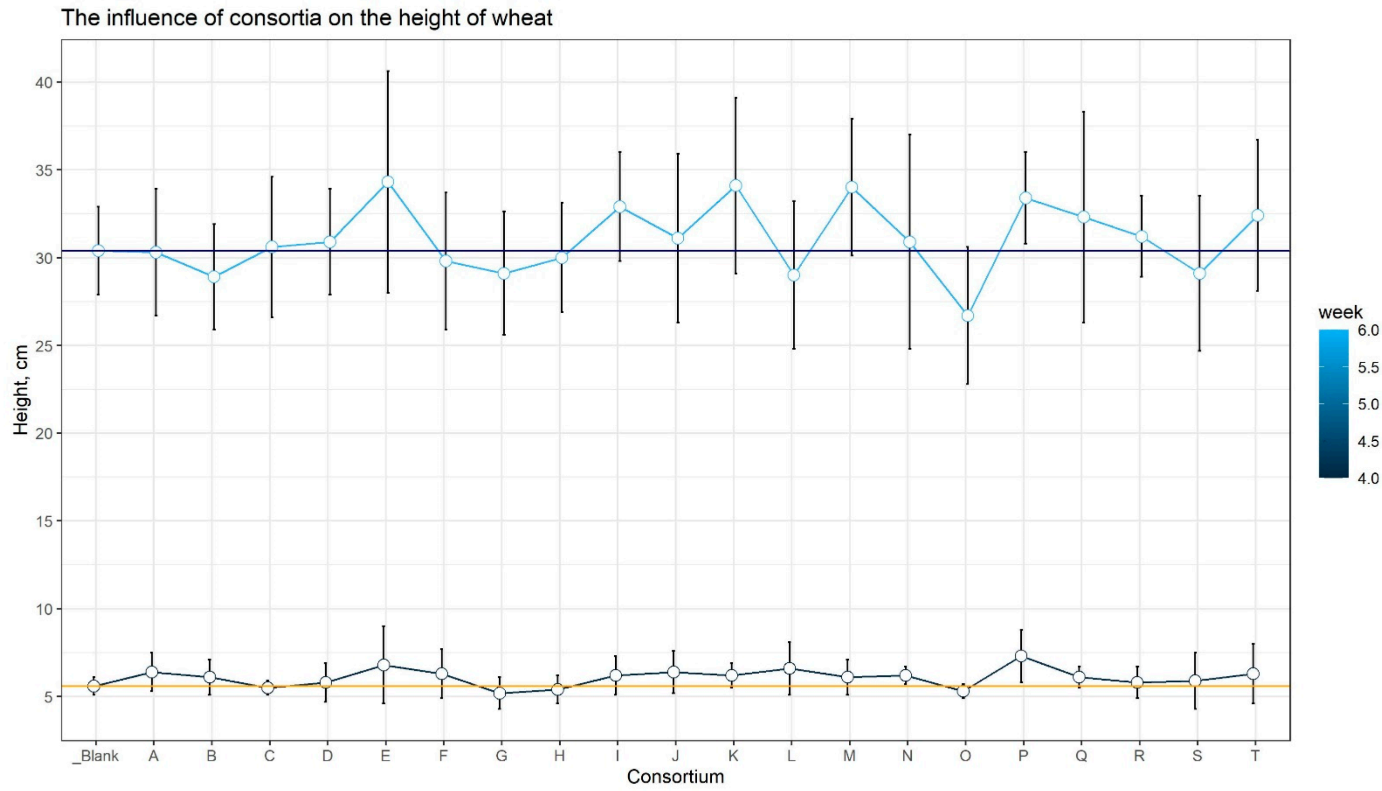

Suppl. Figure S2. Wheat's length of ear and steam of after 3 months.
The red line is corresponding on length of ear, the light blue one – of steam. Orange line corresponds the mean ear's length of control plants, the blue ones the mean and SD of steam's length. One asterisk (*) marks the level of statistical significance of the differences of 0.05 compared to the control plot (Blank).

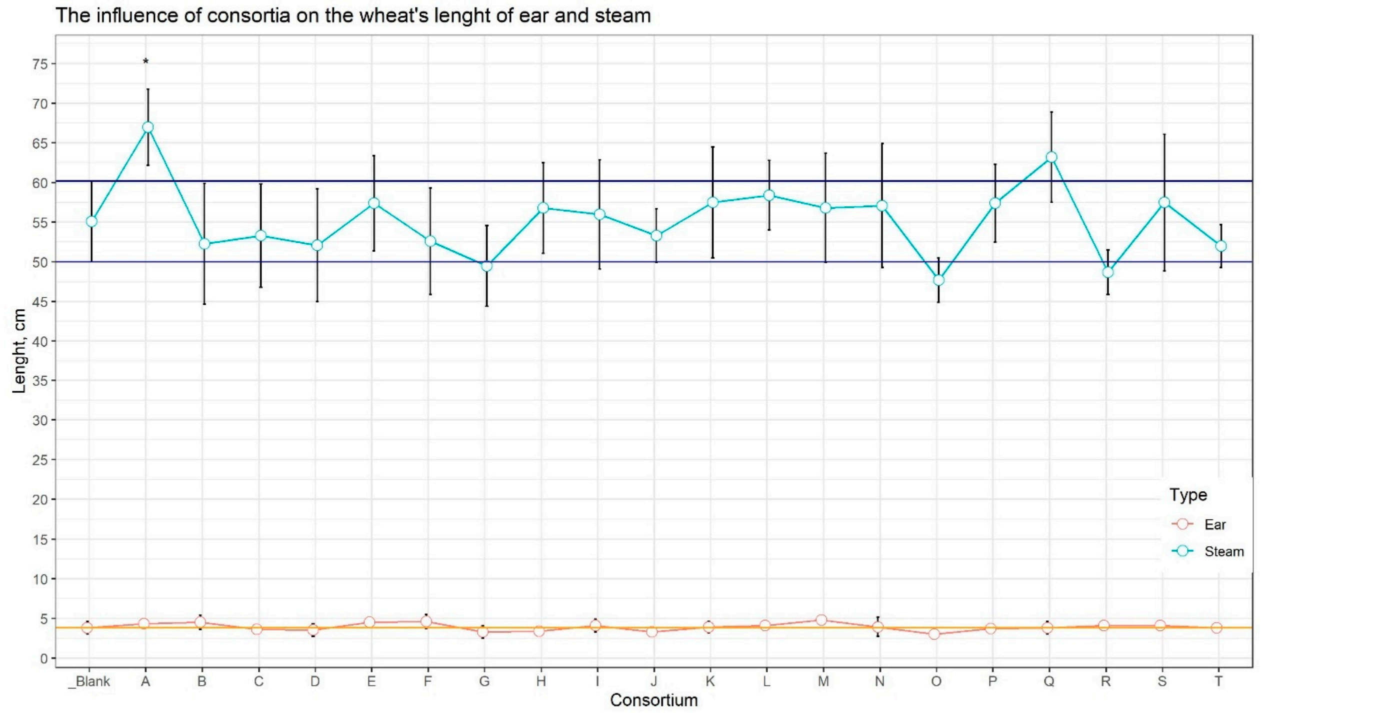

Suppl. Figure S3. Weight of wheat's ear and grain
The red line is corresponding on weight of wheat's ear, the blue points are the weight of grain without a floor.

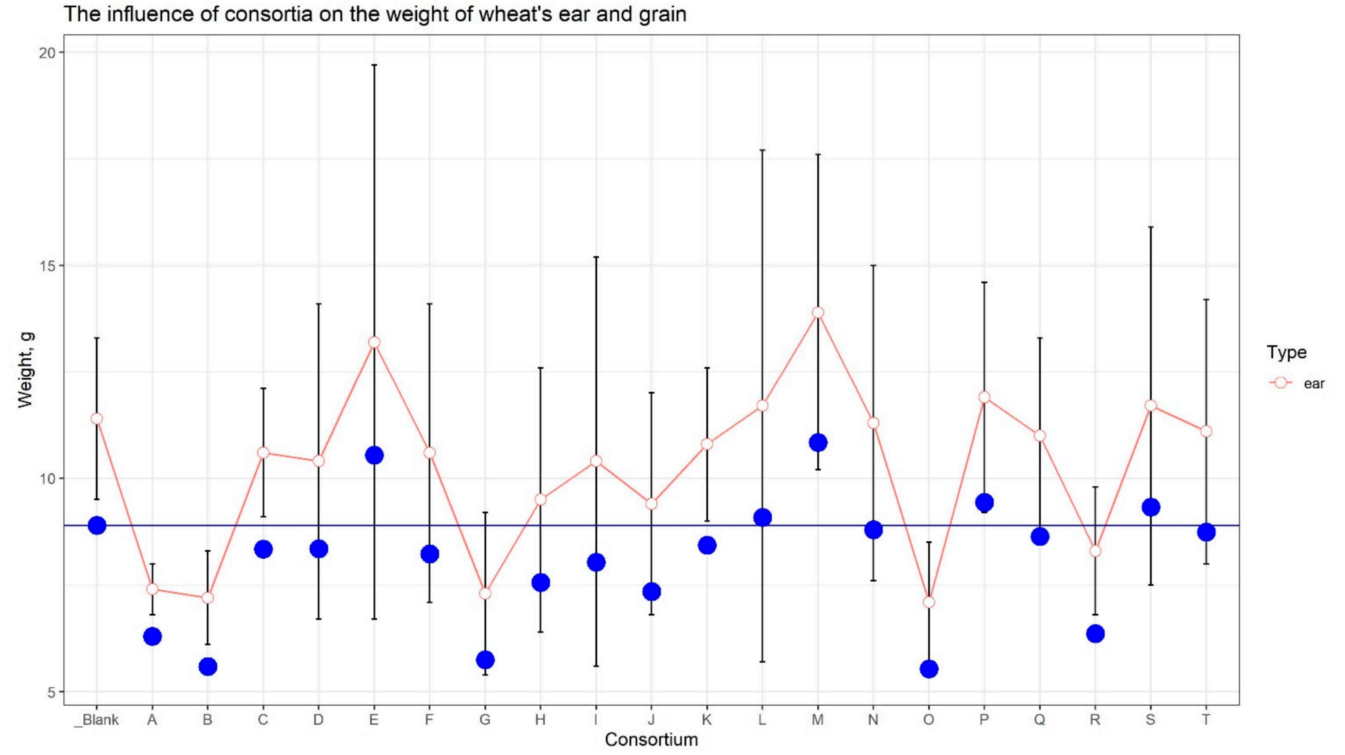

Suppl. Table S4. Corn's yield parameters

Consortium	height of the plants after 4 weeks, cm	height of the plants after 6 weeks, cm	Weight of wet ears of corn, g	Weight of dry ears of corn, g	Weight of dry roots of corn, g
A	9,7 ± 2,5	31,6 ± 7,1	236,7	19,8	551,7
B	8,9 ± 2,9	32,1 ± 8,7	295,0	15,3	481,1
C	9,8 ± 1,2	32,1 ± 4,1	376,7	19,7	577,2
D	9,8 ± 4,1	30,6 ± 10,4	223,3	17,2	387,4
E	10,8 ± 3,5	33,1 ± 8,5	520,0	37,2	690,9
F	8,8 ± 1,4	26,8 ± 5,8	221,7	10,7	412
G	10,6 ± 2,2	27,2 ± 10,8	315,0	29,0	421,9
H	10,5 ± 2,7	30,3 ± 5,7	411,7	30,5	564,1
I	10,0 ± 3,1	36,9 ± 2,7	420,0	22,3	524,4
J	8,5 ± 2,5	27,5 ± 6,8	325,0	22,6	620
K	10,3 ± 2,2	27,3 ± 6,4	301,7	17,7	555,6
L	11,4 ± 2,0	37,4 ± 5,3	366,7	20,9	505,1
M	8,1 ± 2,4	27,3 ± 9,9	348,3	22,5	387,4
N	8,3 ± 2,7	23,1 ± 6,7	181,7	9,9	292,8
O	10,4 ± 2,0	29,1 ± 28,8	173,3	13,7	421,2
P	9,7 ± 2,0	28,8 ± 6,2	338,3	27,8	492,2
Q	9,8 ± 3,4	34,5 ± 7,4	293,3	15,2	386,4
R	9,7 ± 2,1	34,3 ± 5,7	138,3	18,6	558,4
S	8,9 ± 2,0	27,5 ± 8,0	339,0	7,2	238,7
T	10,1 ± 1,6	31,7 ± 7,1	275,0	15,9	630,76
Blank	9,7 ± 2,7	36,5 ± 6,0	521,7	30,2	1133,5

Suppl. Figure S4. Corn's height on 4 and 6 weeks.

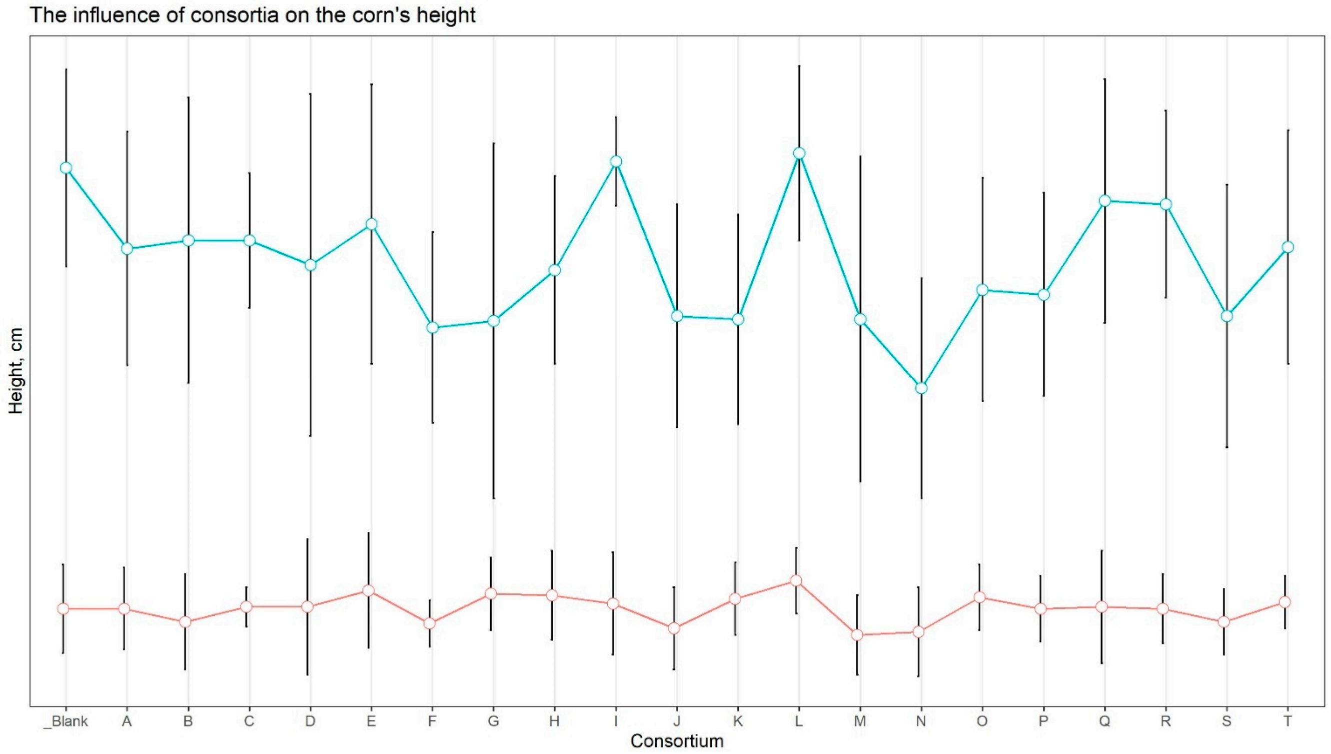

Suppl. Figure S5. Weight of corn's ears and roots
The red line is corresponding on weight of wheat's ear, the blue points are the weight of grain without a floor.

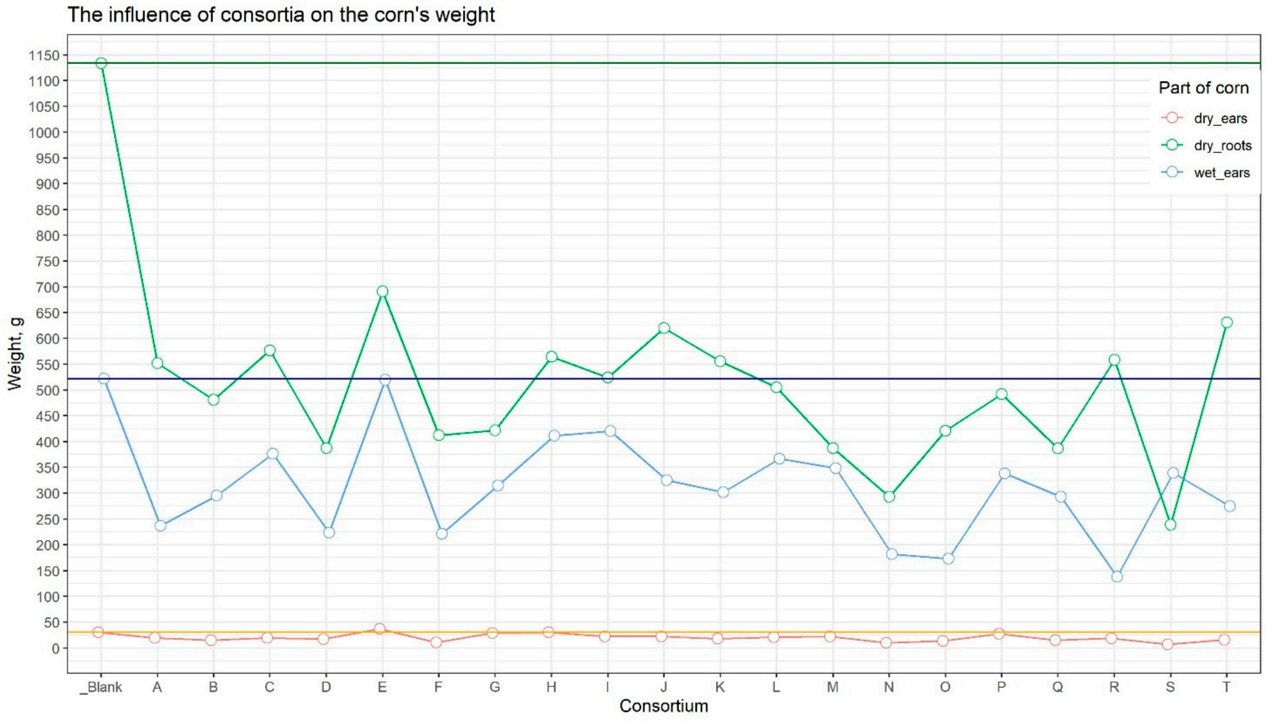

Suppl. Table S5. Soil nutrient status

SIC - soil inorganic carbon, SOC - soil organic carbon, AP - exchangeable potassium, Ex-K - exchangeable potassium, STC - soil total carbon

Two asterisk (**) marks the level of statistical significance of the differences of 0.01 compared to the control plot (Soil).

Cons	AN-NH4	AN-NO3	AP	Ex-K	SOC	SIC	STC
Buckweat							
A	2,35 ± 0,35	4,60 ± 1,38	1,70 ± 0,01	184 ± 18,4	4,40	0,21	4,61
B	1,99 ± 0,30	5,26 ± 1,58	2,14 ±0,024	184 ± 18,4	4,66	0,18	4,84
C	1,55 ± 0,23	4,81 ± 1,44	9,87 ± 0,019**	166 ± 16,6	4,45	0,19	4,64
D	1,12 ± 0,17	5,50 ± 1,65	1,43 ± 0,024	184 ± 18,4	4,81	0,21	5,02
E	1,38 ± 0,21	4,40 ± 1,32	1,66 ± 0,029	184 ± 18,4	4,59	0,18	4,77
F	1,17 ± 0,17	4,81 ± 1,44	1,68 ± 0,005	166 ± 16,6	4,69	0,17	4,87
G	2,01 ± 0,30	5,03 ± 1,51	1,71 ± 0,010	184 ± 18,4	4,61	0,22	4,83
H	4,69 ± 0,70	5,03 ± 1,51	1,59 ± 0,014	184 ± 18,4	4,41	0,20	4,61
I	3,69 ± 0,55	4,81 ± 1,44	1,32 ± 0,010	166 ± 16,6	4,71	0,17	4,89
J	4,58 ± 0,69	5,26 ± 1,58	1,23 ± 0,024	166 ± 16,6	4,34	0,20	4,54
K	5,07 ± 0,76	5,26 ± 1,58	3,04 ± 0,00	166 ± 16,6	4,33	0,18	4,50
L	2,46 ± 0,37	4,81 ± 1,44	1,59 ± 0,010	150 ± 15,0	4,51	0,19	4,70
M	3,29 ± 0,49	4,81 ± 1,44	1,63 ± 0,005	166 ± 16,6	4,37	0,19	4,57
N	3,75 ± 0,56	4,81 ± 1,44	1,76 ± 0,010	166 ± 16,6	4,61	0,19	4,81
O	2,31 ± 0,35	4,21 ± 1,26	1,18 ± 0,029	150 ± 15,0	4,69	0,20	4,89
P	1,57 ± 0,24	4,40 ± 1,32	1,52 ± 0,00	166 ± 16,6	4,37	0,19	4,57
R	2,16 ± 0,32	4,40 ± 1,32	1,58 ± 0,014	150 ± 15,0	4,34	0,22	4,56
S	2,40 ± 0,36	4,40 ± 1,32	1,56 ± 0,014	166 ± 16,6	4,54	0,20	4,73
T	1,95 ± 0,29	4,21 ± 1,26	1,22 ± 0,010	150 ± 15,0	4,77	0,20	4,97
Q	2,16 ± 0,32	4,60 ± 1,38	1,51 ± 0,010	184 ± 18,4	4,71	0,18	4,89
Blank	5,30 ± 0,80	5,26 ± 1,58	1,91 ± 0,000	184 ± 18,4	4,66	0,19	4,85
Soil	3,51 ± 0,53	4,03 ± 1,21	1,01 ± 0,024	134 ± 13,4	4,68	0,22	4,91
Wheat							
A	1,99 ± 0,30	6,05 ± 1,82	0,82 ± 0,009	165 ± 16,5	4,89	0,18	5,07
B	2,06 ± 0,31	5,02 ± 1,51	0,64 ± 0,014	175 ± 17,5	4,86	0,19	5,06
C	2,57 ± 0,38	4,57 ± 1,37	1,01 ± 0,005	170 ± 17,0	4,64	0,18	4,82
D	1,93 ± 0,29	4,16 ± 1,25	0,62 ± 0,019	160 ± 16,0	4,41	0,18	4,58
E	2,44 ± 0,37	5,02 ± 1,51	0,86 ± 0,014	175 ± 17,5	4,67	0,17	4,84
F	2,99 ± 0,45	3,97 ± 1,19	0,72 ± 0,024	155 ± 15,5	4,12	0,18	4,30
G	0,95 ± 0,14	3,97 ± 1,19	0,59 ± 0,00	160 ± 16,0	4,00	0,20	4,21
H	1,44 ± 0,22	4,57 ± 1,37	1,05 ± 0,005	180 ± 18,0	4,74	0,19	4,93
I	0,64 ± 0,10	4,36 ± 1,31	0,69 ± 0,019	170 ± 17,0	4,66	0,18	4,84
J	0,72 ± 0,11	4,36 ± 1,31	0,58 ± 0,009	160 ± 16,0	4,17	0,17	4,34
K	1,84 ± 0,28	3,97 ± 1,19	0,72 ± 0,024	175 ± 17,5	4,67	0,18	4,85
L	0,64 ± 0,10	4,79 ± 1,44	1,03 ± 0,024	206 ± 20,6	4,61	0,18	4,79
M	1,55 ± 0,23	3,79 ± 1,14	0,63 ± 0,019	165 ± 16,5	4,55	0,19	4,74
N	0,98 ± 0,15	4,16 ± 1,25	0,88 ± 0,005	165 ± 16,5	4,16	0,25	4,41
O	1,27 ± 0,19	3,97 ± 1,19	1,00 ± 0,009	175 ± 17,5	4,70	0,17	4,87
P	4,85 ± 0,73	3,97 ± 1,19	1,21 ± 0,00	175 ± 17,5	4,64	0,19	4,82
Q	2,12 ± 0,32	3,97 ± 1,19	0,72 ± 0,005	165 ± 16,5	4,63	0,20	4,83
R	1,97 ± 0,30	3,97 ± 1,19	0,69 ± 0,019	180 ± 18,0	4,50	0,18	4,68
S	1,34 ± 0,20	3,62 ± 1,09	0,80 ± 0,024	180 ± 18,0	4,09	0,20	4,29

T	3,18 ± 0,48	4,16 ± 1,25	0,75 ± 0,024	165 ± 16,5	4,07	0,16	4,23
Blank	2,12 ± 0,32	3,97 ± 1,19	1,11 ± 0,009	165 ± 16,5	4,52	0,17	4,69
Soil	3,40 ± 0,51	3,97 ± 1,19	0,65 ± 0,009	150 ± 15,0	3,96	0,17	4,13
Corn							
A	0,95 ± 0,14	6,34 ± 1,90	0,40 ± 0,009	112 ± 11,2	2,11	0,13	2,24
B	1,44 ± 0,22	7,30 ± 2,19	0,28 ± 0,014	120 ± 12,0	3,22	0,15	3,37
C	0,76 ± 0,11	6,65 ± 1,99	0,28 ± 0,005	124 ± 12,4	3,03	0,14	3,17
D	0,21 ± 0,03	5,02 ± 1,51	0,21 ± 0,024	137 ± 13,7	2,97	0,14	3,11
E	1,34 ± 0,20	6,34 ± 1,90	0,25 ± 0,005	124 ± 12,4	3,24	0,16	3,40
F	0,83 ± 0,12	5,78 ± 1,73	0,35 ± 0,009	116 ± 11,6	3,13	0,15	3,28
G	1,78 ± 0,27	5,51 ± 1,65	0,25 ± 0,014	108 ± 10,8	2,57	0,14	2,72
H	1,51 ± 0,23	5,02 ± 1,51	0,32 ± 0,014	112 ± 11,2	2,41	0,16	2,58
I	1,06 ± 0,16	5,78 ± 1,73	0,29 ± 0,009	165 ± 16,2	2,97	0,17	3,14
J	0,66 ± 0,10	5,78 ± 1,73	0,31 ± 0,005	120 ± 12,0	2,82	0,30	3,12
K	1,34 ± 0,20	6,05 ± 1,82	0,37 ± 0,000	96 ± 48,0	2,69	0,13	2,82
L	1,97 ± 0,30	4,79 ± 1,44	0,23 ± 0,009	137 ± 13,7	2,80	0,17	2,97
M	1,95 ± 0,29	4,79 ± 1,44	0,25 ± 0,009	100 ± 10,0	2,51	0,12	2,63
N	2,14 ± 0,32	4,57 ± 1,37	0,38 ± 0,014	87 ± 13,1	2,96	0,15	3,11
O	1,19 ± 0,18	4,79 ± 1,44	0,27 ± 0,009	129 ± 12,9	2,32	0,11	2,43
P	1,51 ± 0,23	5,02 ± 1,51	0,31 ± 0,024	150 ± 15,0	3,12	0,14	3,27
R	1,25 ± 0,19	4,16 ± 1,25	0,27 ± 0,000	124 ± 12,4	2,61	0,14	2,75
S	0,28 ± 0,04	5,78 ± 1,73	0,28 ± 0,005	120 ± 12,0	2,81	0,14	2,95
T	0,81 ± 0,12	4,36 ± 1,31	0,34 ± 0,014	146 ± 14,6	3,10	0,18	3,28
Q	0,91 ± 0,14	4,79 ± 1,44	0,25 ± 0,019	146 ± 14,6	3,43	0,17	3,60
Blank	1,51 ± 0,23	5,02 ± 1,51	0,36 ± 0,005	116 ± 11,6	2,88	0,14	3,03
Soil	1,38 ± 0,21	6,05 ± 1,82	0,37 ± 0,000	108 ± 10,8	2,95	0,14	3,10

Suppl. Figure S6. Dependence of detection of the number of taxa (OTUs) on the number of sequences (N). The dependence of the detection of the number of taxa (OTU) on the number of sequences (N). The x-axis shows the number of sequences, the ordinate axis shows the observed species (OTU). The curves on the graph correspond to all the soil samples studied. As the sequencing depth increased, so did the number of OTU. After 700 sequences, the curves for all samples reached a plateau, indicating that as the number of extracted sequences increased, the number of detectable OTU no longer increased.

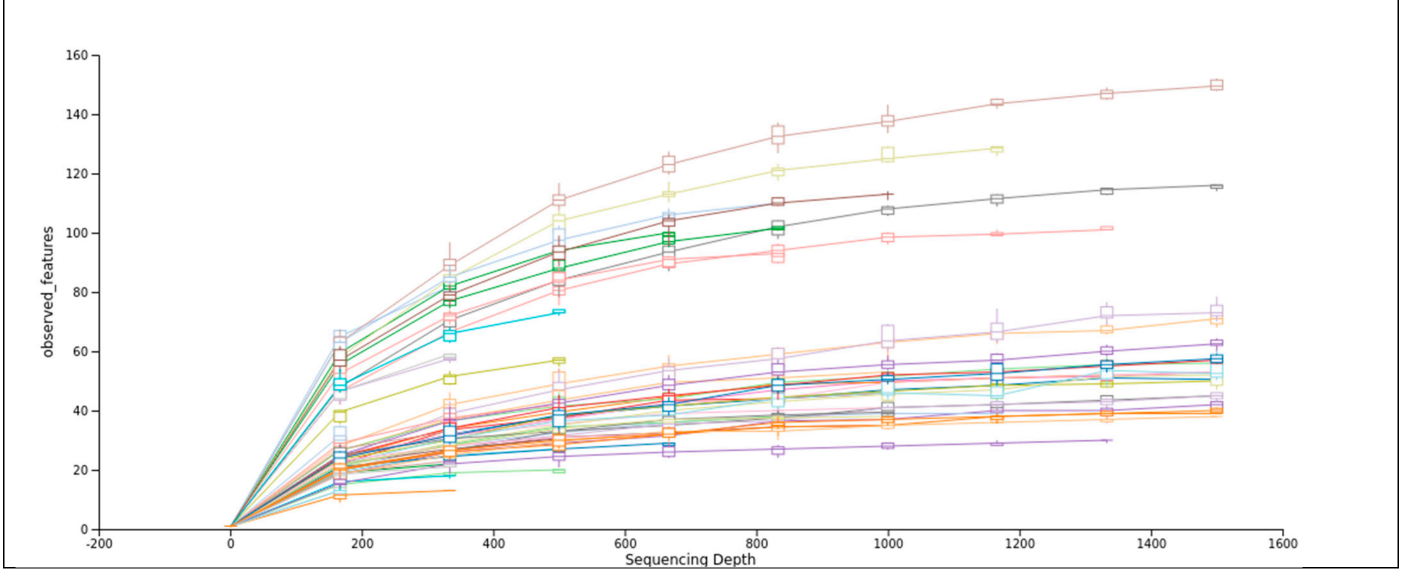

Suppl. Table S6. Statistical table of α-diversity index under different consortium on wheat, corn and гречихи.

Consortsium	Obs	Chao1	ACE	Shannon
A	40,5 ± 38,9	62,2 ± 49,4	61,5 ± 48,2	2,51 ± 1,50
B	42,3 ± 28,4	65,5 ± 46,2	67,8 ± 49,2	2,64 ± 0,97
C	34 ± 13,5	56,5 ± 31,5	79,5 ± 51,6	2,30 ± 0,51
D	38,3 ± 25,7	63,3 ± 47,1	71,9 ± 53,6	2,43 ± 0,75
E	31,7 ± 25,8	58,2 ± 63,9	58,2 ± 62,9	2,27 ± 0,67
F	40,7 ± 23,0	64,1 ± 51,9	72,9 ± 61,6	2,50 ± 0,69
G	25,7 ± 3,8	32,0 ± 9,8	35,4 ± 11,9	2,12 ± 0,16
H	23,7 ± 4,0	29,8 ± 8,7	58,3 ± 7,7	2,11 ± 0,12
I	31,7 ± 6,0	50,1 ± 7,0	61,8 ± 12,3	2,23 ± 0,46
J	35,0 ± 11,1	53,6 ± 7,9	30,9 ± 11,4	2,39 ± 0,46
K	23,0 ± 5,7	29,0 ± 12,8	44,0 ± 21,1	2,03 ± 0,04
L	32,0 ± 15,9	40,0 ± 18,7	29,7 ± 11,6	2,36 ± 0,70
M	21,5 ± 3,54	26,4 ± 9,3	63,8 ± 51,5	1,95 ± 0,06
N	43,5 ± 34,6	54,6 ± 44,4	57,1 ± 40,7	2,72 ± 1,28
O	35,7 ± 22,0	53,1 ± 42,6	57,1 ± 40,7	2,31 ± 0,93
P	34,0 ± 20,2	52,9 ± 35,6	59,1 ± 38,2	2,36 ± 0,73
Q	36,3 ± 17,9	66,8 ± 40,4	70,9 ± 44,3	2,42 ± 0,56
S	21,0 ± 5,7	32,1 ± 21,0	29,8 ± 16,3	1,92 ± 0,06
T	25,7 ± 4,5	36,7 ± 13,7	39,6 ± 13,7	2,18 ± 0,24
Soil	27,3 ± 16,9	34,3 ± 28,7	36,5 ± 32,2	2,28 ± 0,74
Blank	31,0 ± 12,7	45,0 ± 12,7	57,1 ± 9,6	2,25 ± 0,18
Plant	Obs	Chao1	ACE	Shannon
bw	27,5 ± 9,1	41,9 ± 19,6	48,9 ± 27,1	2,18 ± 0,37
corn	46 ± 18,8	73,1 ± 37,8	80,3 ± 39,4	2,78 ± 0,64
wheat	22,85 ± 6,5	30,8 ± 12,8	33,1 ± 13,8	1,98 ± 0,26

Suppl. Figure S7. Box plot of indices of alpha diversity of microorganisms in soil plots. From A to T — different types of consortia. Blank is a soil sample on which seeds not inoculated with microorganisms grew. Soil - a soil sample on which the tested plant culture did not grow

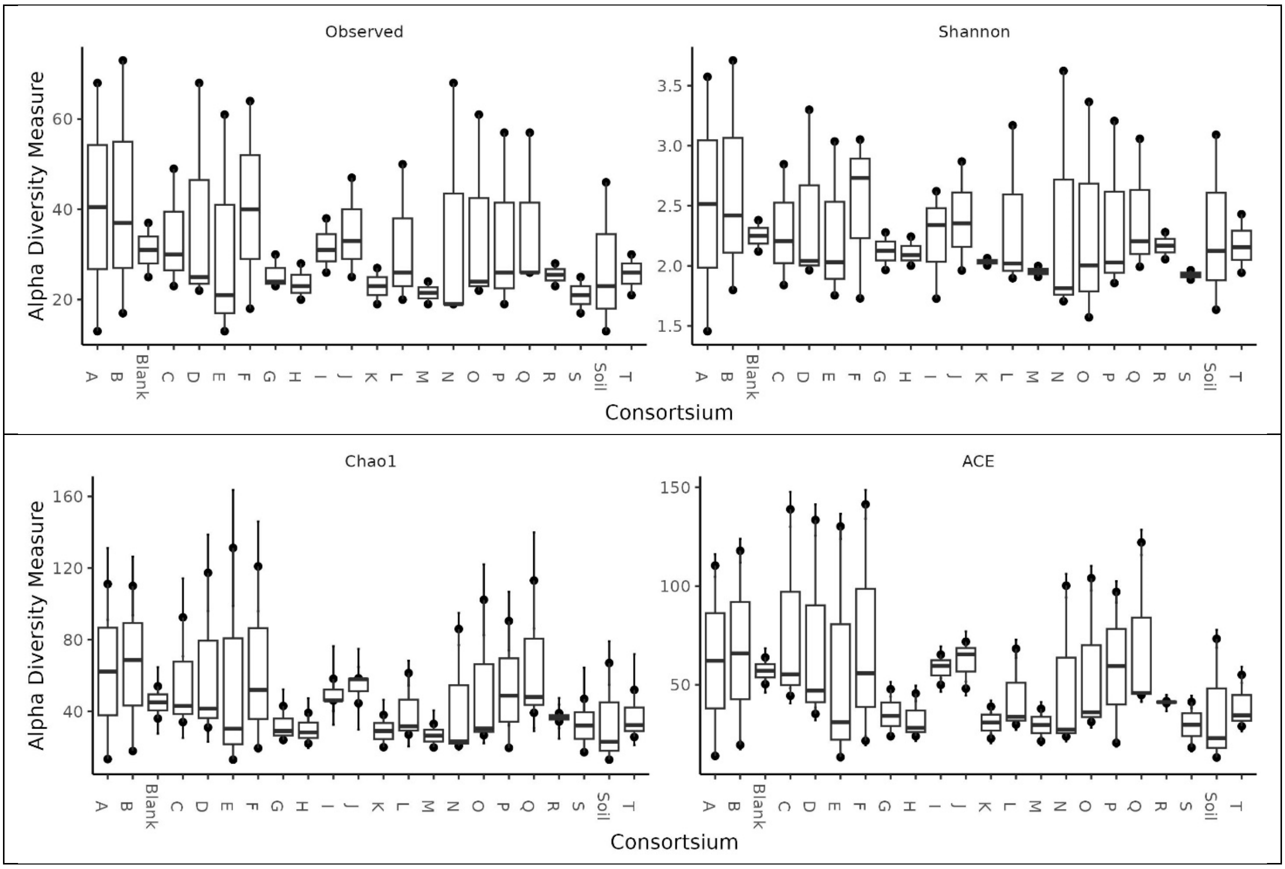

Observed
Shannon
Chao1
ACE
Alpha Diversity Measure
Consortsium
A
B
Blank
C
D
E
F
G
H
I
J
K
L
M
N
O
P
Q
R
S
Soil
T
20
40
60
1.5
2.0
2.5
3.0
3.5
40
80
120
160
50
100
150
